# Evolutionary insights into Felidae iris color through ancestral state reconstruction

**DOI:** 10.1016/j.isci.2024.110903

**Published:** 2024-09-13

**Authors:** Julius A. Tabin, Katherine A. Chiasson

**Affiliations:** 1 Department of Organismic and Evolutionary Biology, Harvard University, Cambridge, MA 02138, USA; 2 Krieger School of Arts and Sciences, Johns Hopkins University, Baltimore, MD 21218, USA

**Keywords:** Evolutionary Biology, Zoology, Phylogeny

## Abstract

Few studies have explored eye (iris) color evolution beyond humans and domesticated animals. Felids exhibit significant eye color diversity, unlike their brown-eyed relatives, making them an ideal model to study the evolution of eye color in natural populations. Through machine learning analysis of public photographs, five felid eye colors were identified: brown, green, yellow, gray, and blue. The presence or absence of these colors was reconstructed on a phylogeny, as well as their specific quantitative shades. The ancestral felid population likely had brown-eyed and gray-eyed individuals, the latter color being pivotal for the diversification of eye color seen in modern felids. Additionally, yellow eyes are highly associated with and may be necessary for, the evolution of round pupils in felids. These findings enhance the understanding of eye color evolution, and the methods presented in this work are widely applicable and will facilitate future research into the phylogenetic reconstruction of color beyond irises.

## Highlights

• Five eye colors were identified for the Felidae: brown, green, yellow, gray, and blue
• The ancestral felid population likely had both brown-eyed and gray-eyed individuals
• The evolution of gray eyes may have led to the evolution of other eye colors
• Eye color evolution can be effectively studied using digital image analysis methods

## Graphical abstract

**Figure:**
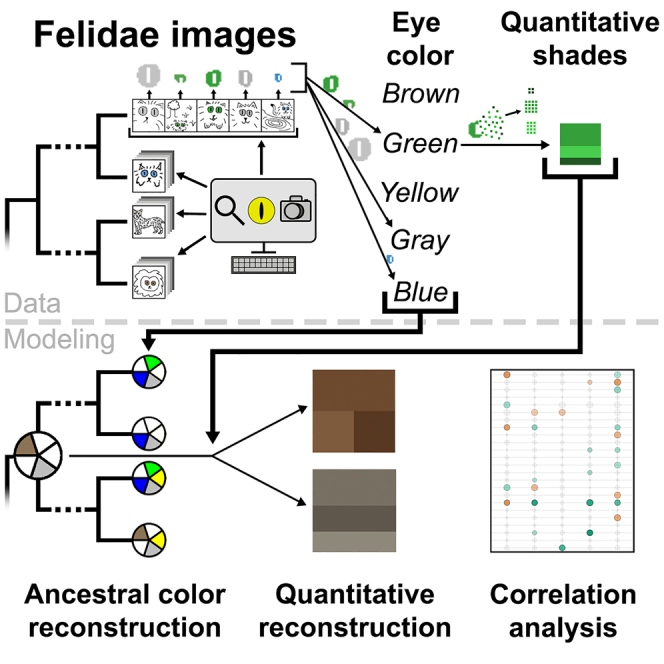

## Introduction

Eye (iris) color is one of the most conspicuous and varied traits among animals. To date, much of the work investigating eye colors has focused on humans. This is not surprising, given how stark differences in human eye color can be, even between close relatives. This diversity of human eye colors, ranging from brown to green to blue, has been suggested to not be under strong natural selection.^1^ Thus, it has been attributed to bottlenecks and genetic drift, migration,^2^ and especially sexual selection.^3^ It is known that human eye colors differ due to a relatively small number of genes that act on the amount, type, and quality of melanin in the eye.^4^^,^^5^ Yet, such eye color variation within a species (intraspecific variation) has been described as rare amongst animals, apart from artificially selected domesticated animals and some species of birds.^6^ This latter variation has been mainly attributed to age, sex, or subspecies identity.^7^

In humans, the discrete view of intraspecific variation in eye color has also been challenged by more continuous models of variation.^8^ Traditional categories, such as blue, green, and brown, have been shown to overlap to varying degrees. This problem has been demonstrated for other color traits as well: for example, the colors of snail shells,^9^ tree lizard throats,^10^ and bird plumage.^11^ These studies indicate that, although discrete categories are useful for trait comprehension and can facilitate clear analyses, results can be compromised if the categories do not reflect the true trait distribution. Some studies advocate for coupling analysis of discrete categories with more granular analysis within individuals of a category.^11^ Comprehensive investigation into the validity and utility of discrete categories for eye color has been lacking, especially since the optimal way to categorize color variation is still debated even for more well-studied traits (e.g.,^12^^,^^13^). In this way, not only has there been limited evidence for intraspecific variation in eye color beyond humans and domesticants, but it has also been unclear how to best categorize that variation, should it exist.

Even when just regarding eye color differences between species (interspecific variation), few hypotheses have been tested and very little is known about the adaptive benefits or evolutionary history of eye color, particularly in a natural context. While a few studies have attempted to tackle these questions by focusing primarily on distinctions between brightly or darkly colored irises, this method eschews quantitative color measurements and limits the conclusions that can be drawn.^14^ It is of particular interest to reconstruct the ancestral state of eye color because such reconstructions can shed light on the history of the trait and help interrogate how and why the current variants exist. Such analyses are vital for broadly increasing knowledge of evolution in a natural context, especially since eye color is not retained in fossils, nor in most preserved specimens. This has been done to great effect in owls; however, only “light” and “dark” eyes were considered in the analysis, not specific eye colors.^15^

**Figure 1:**
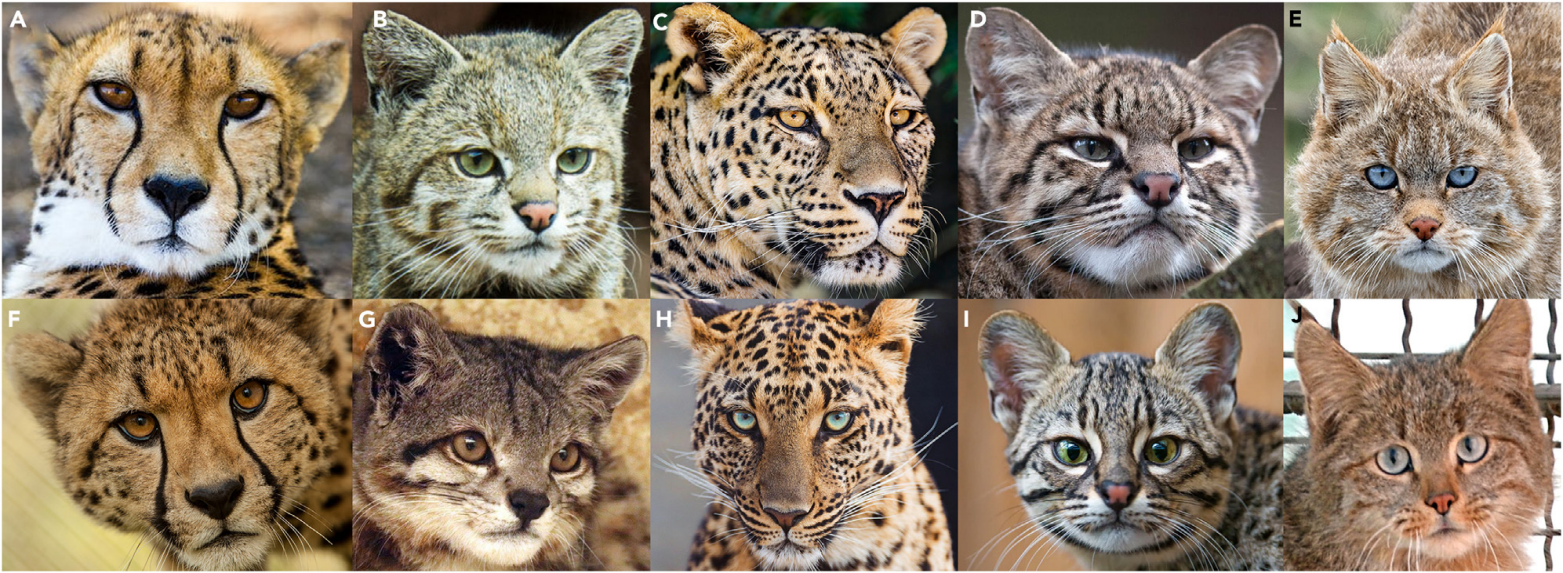
Examples of the five felid eye colors, with inter- and intraspecific variation Each row contains an example of each of the five eye colors and each column contains two representatives of the same species. Colors and species are as follows: (A) brown (*Acinonyx jubatus*), (B) green (*Leopardus jacobita*), (C) yellow (*Panthera pardus*), (D) gray (*Leopardus geoffroyi*), (E) blue (*Felis bieti*), (F) yellow (*Acinonyx jubatus*), (G) brown (*Leopardus jacobita*), (H) blue (*Panthera pardus*), (I) green (*Leopardus geoffroyi*), (J) gray (*Felis bieti*). Photographs from (A) Piet Bakker, Pexels, (B) Luis D Romero, Shutterstock, (C) Tambako The Jaguar, Flickr, (D) www.dinoanimals.com, (E) Song Dazhao, CFCA, (F) Ronda Gregorio, Smithsonian National Zoo, (G) Nayer Youakim, (H) Tambako The Jaguar, Flickr, (I) Tambako The Jaguar, Flickr, (J) Abujoy, Wikimedia Commons.

As with most groups, little work has been done to understand the eye colors of members of the family Felidae. Behavioral traits have been correlated to the eye colors of domesticated cats (*Felis catus*), but the wild felids have been largely left unstudied in this regard.^16^ This is surprising – although the closest relatives to the felids, such as linsangs, hyenas, and genets,^17^ all have brown eyes and little inter- or intraspecific variation, the felids have a wide diversity of eye colors within and between them, even without counting *F. catus* (Figure 1). Although this trait is apparent by simply looking at members of each species, it has never been studied in an evolutionary context.

Here, we present the first quantitative phylogenetic comparative analysis of eye color. We examine representatives from every extant felid species, as well as a number of subspecies, using a new quantitative color analysis method to solidify a categorization of eye color for these groups through both discrete and continuous methods. Using this data, we have reconstructed the eye colors of the ancestors of the felids at all phylogenetic nodes.

We also correlated these eye color results with environmental, behavioral, and morphological data. Felids are distributed worldwide, naturally present on every continent except Antarctica and Oceania, and occupy a wide diversity of habitats, such as tropical rainforests, deserts, and mountainous regions. Due to the potential for eye color to be distributed in a non-random way across these broad environments, we investigated the influence of zoogeographical region and habitat.^18^ For behavioral data, we focused on activity mode, since felid species vary in whether they are nocturnal, crepuscular, and/or diurnal, and previous work has suggested a link between nocturnality and the shade of eyes in owls.^15^ Additionally, given the role of melanin in determining eye color, as well as the varied interspecific body pigmentation phenotypes in the felids, we investigated correlations of eye colors with coat patterns, coat pigmentation, and nose pigmentation, with the latter being a proxy for skin color. Lastly, felids can have either round or vertical pupils and, given the pupil’s proximity to the iris and the potential for interactions between the two structures, we explored this trait’s correlation with eye color as well. Overall, our analysis allows us to better understand the diversification of felid eye colors and demonstrates that phylogenetic investigations into eye color are not only possible, but fruitful.

## Results

### Inter- and intraspecific felid eye color variation

To assess the range of eye colors present in the Felidae, we leveraged high-quality public image databases and sampled individuals from all non-domesticated felid species, as well as four related outgroups (see STAR Methods for details). Which eye colors are present for each taxon was determined impartially using color identification software (STAR Methods). Within the 52 felid taxa considered in the study, gray eyes were found to be present in 38 taxa (73%), brown eyes in 28 taxa (54%), yellow eyes in 23 taxa (44%), green eyes in 21 taxa (40%), and blue eyes in 6 taxa (12%). These statistics are given in Table 1, along with the results when subspecies are not considered and when just the most common eye colors (eye colors present in a plurality of each taxon’s individuals in the surveyed databases; see STAR Methods for exact thresholds) are considered. Figure 1 contains representative images for each identified eye color.

**Table 1:** Eye color analysis count results

Eye Color	In All Felid Taxa	Without Subspecies	Most Common Colors in All Felid Taxa	Most Common Colors Without Subspecies
Gray	38 (73%)	31 (72%)	31 (60%)	23 (53%)
Brown	28 (54%)	28 (65%)	22 (42%)	22 (51%)
Yellow	23 (44%)	17 (40%)	16 (31%)	10 (23%)
Green	21 (40%)	20 (47%)	11 (21%)	10 (23%)
Blue	6 (12%)	5 (13%)	2 (4%)	2 (5%)

**Figure 2:**
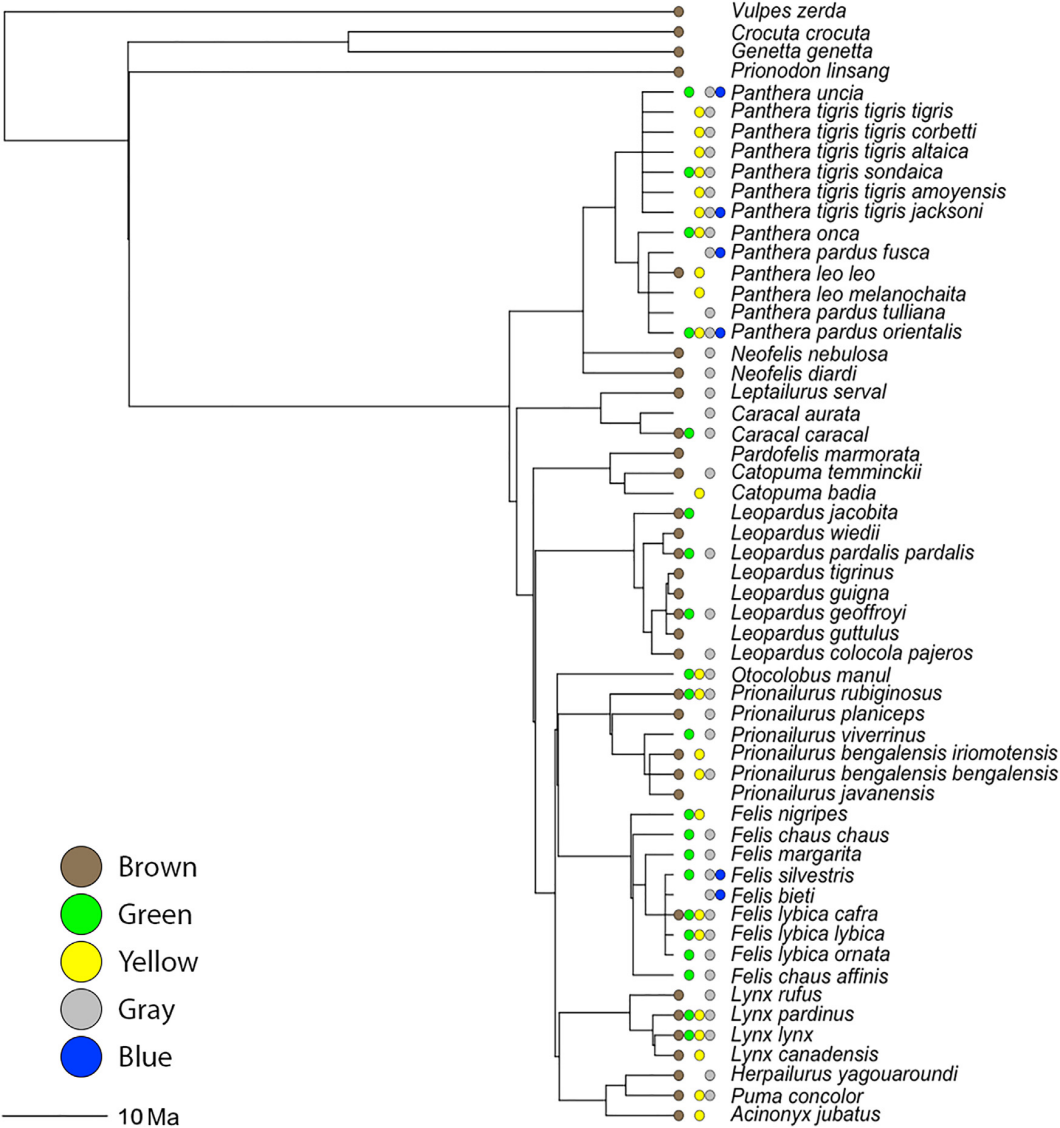
Phylogenetic tree of the Felidae (and outgroups), modified from Nyakatura and Bininda-Emonds (2012), with iris color data Dots next to tips represent the presence of each eye color (brown dot: brown eyes, green dot: green eyes, yellow dot: yellow eyes, gray dot: gray eyes, blue dot: blue eyes). A lack of a dot for a given color represents an absence of that color from the respective taxa. Graph created using plot.phylo() in ape.

In 10 felid taxa only a single eye color was observed, in 25 taxa two eye colors were observed, in 12 three eye colors were observed, and in 5 four eye colors were observed. All the eye colors present for each species are displayed in Figure 2. When considering just the most common eye colors in the populations of each taxon, 25 felid taxa had only a single eye color, 24 taxa had two eye colors, and 3 had three eye colors. Even with this conservative filtering of the data, there is conclusive evidence of the presence of intraspecific iris color variation among the Felidae.

### Eye color polymorphism assessment

**Figure 3:**
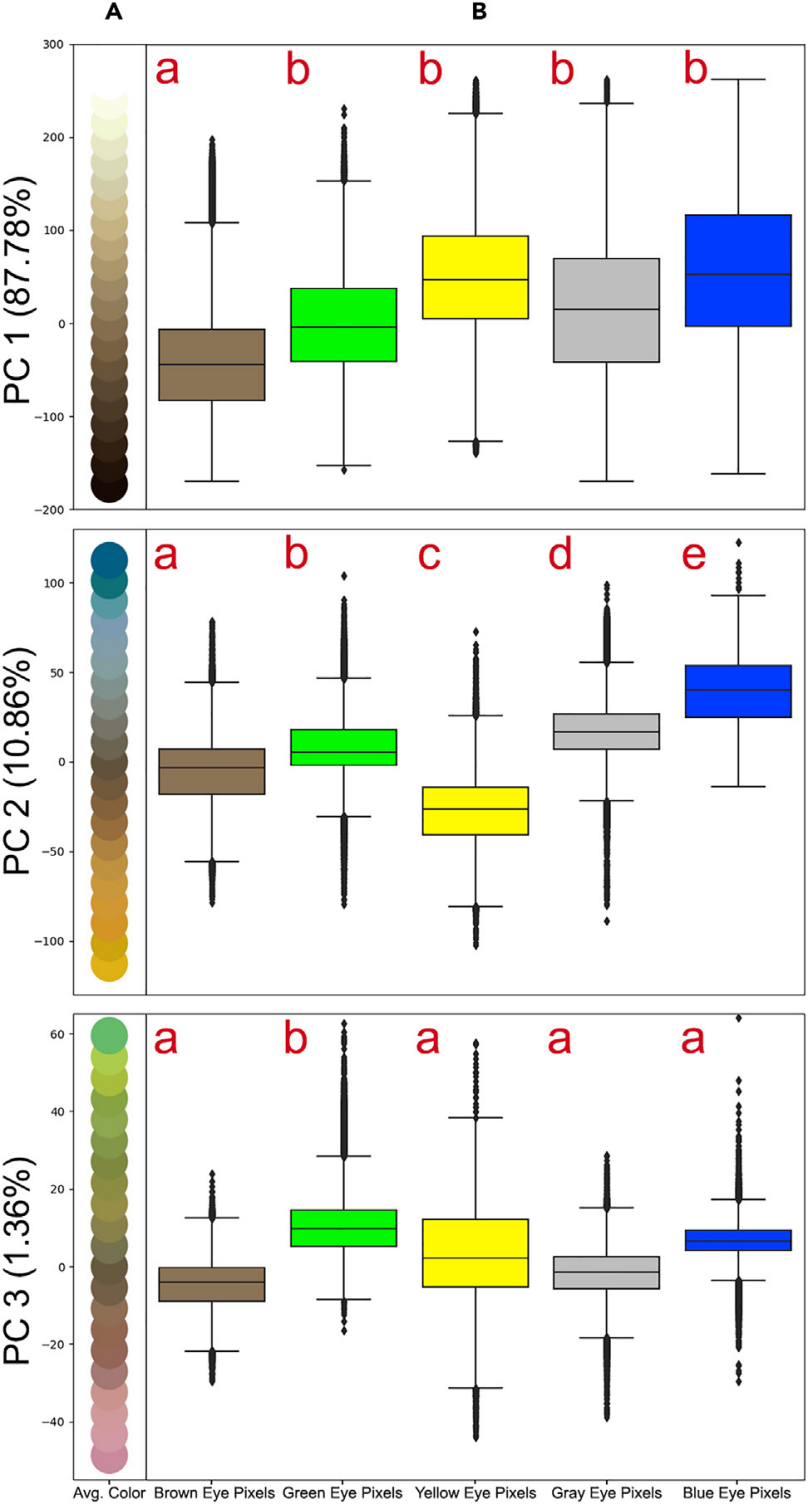
Results of a principal component analysis on the RGB values for all pixels in the dataset (A) The average pixel color along PCs 1–3. The color average presented was calculated for the same data in 20 equally spaced bins along each PC. (B) Distribution of pixels from all eyes in the dataset for each of the color categories for each PC. Different letters indicate groups with significant differences (*p* < 0.05) according to the post-hoc Tukey HSD test, after comparing linear mixed models with Satterthwaite’s t-test, both tests with Bonferroni correction. Data are represented as IQR +/− 1.5∗IQR.

The adequacy of analyzing felid eye color as a discrete polymorphic trait was supported by a principal component analysis on the RGB values of all the pixels in the dataset (STAR Methods; Figure S1). Principal component (PC) 1 was found to separate dark from light pixel coloration (Figure 3) and only brown eyes were found to be a meaningfully distinct category along that axis (*p* < 0.001, Satterthwaite’s t-test on a linear mixed model with Bonferroni correction; Table S1). In other words, the dataset photos of brown eyes contained significantly darker pixels than the other colors, unsurprising given their darker pigment. While pigment darkness is a biologically relevant metric for eyes,^15^ this axis is not sufficient to judge the biological validity of discrete color categories, since it only encapsulates a singular aspect of color variation. Two eyes with unambiguously distinct colors could still have the same darkness value. An axis that directly contains the colors of interest is far more relevant to determine whether the categories are significantly separate: if one wants to evaluate whether blue and gray, for instance, are different categories in the dataset, the axis of measurement must thus directly contain blue and gray.

PC2 was found to separate blue, gray, brown, and yellow pixels and all color categories were significant along that axis (*p* < 0.0001 for all groups, as well as for all group comparisons, Satterthwaite’s t-test on a linear mixed model with Bonferroni correction and a post-hoc Tukey HSD test with Bonferroni correction, respectively; Table S1). There was little overlap between the pixels within the categories along PC2, except for green, which overlaps with brown and gray. The distinctness and separate modality of the pixel distributions for each color along PC2 can be seen in Figure S2, with each color containing a distinct pixel density peak. Although there seems to be some overlap between color categories, any two categories are clearly distinct. For example, although the two main peaks for brown are under the yellow and green peaks respectively, this does not mean that yellow and green are subsets of brown or vice versa. It means that any given brown eye may have some pixels that have similar values along PC2 as yellow eyes, as well as some pixels that have similar values as green eyes.

PC3 was found to separate green pixels from other pixel colors. In this case, only green eyes were found to be meaningfully different along the axis (*p* < 0.001, Satterthwaite’s t-test on a linear mixed model with Bonferroni correction). In sum, the first three PCs comprised 100% of the variance in pixel coloration. Thus, for the dataset as a whole, the presence of the five discrete color groups was supported and those color groups were able to be easily distinguished along relevant axes. Support for the discrete color categories was also found for every species in the dataset when analyzed individually along PC 2 (STAR Methods), except for *Acinonyx jubatus* (*p* = 1.00, Kruskal–Wallis test with Bonferroni correction; Figure S3; Table S1). Images of color variation in *Acinonyx jubatus* can be seen in Figures 1A and 1F.

### General ancestral state reconstruction of eye color

**Figure 4:**
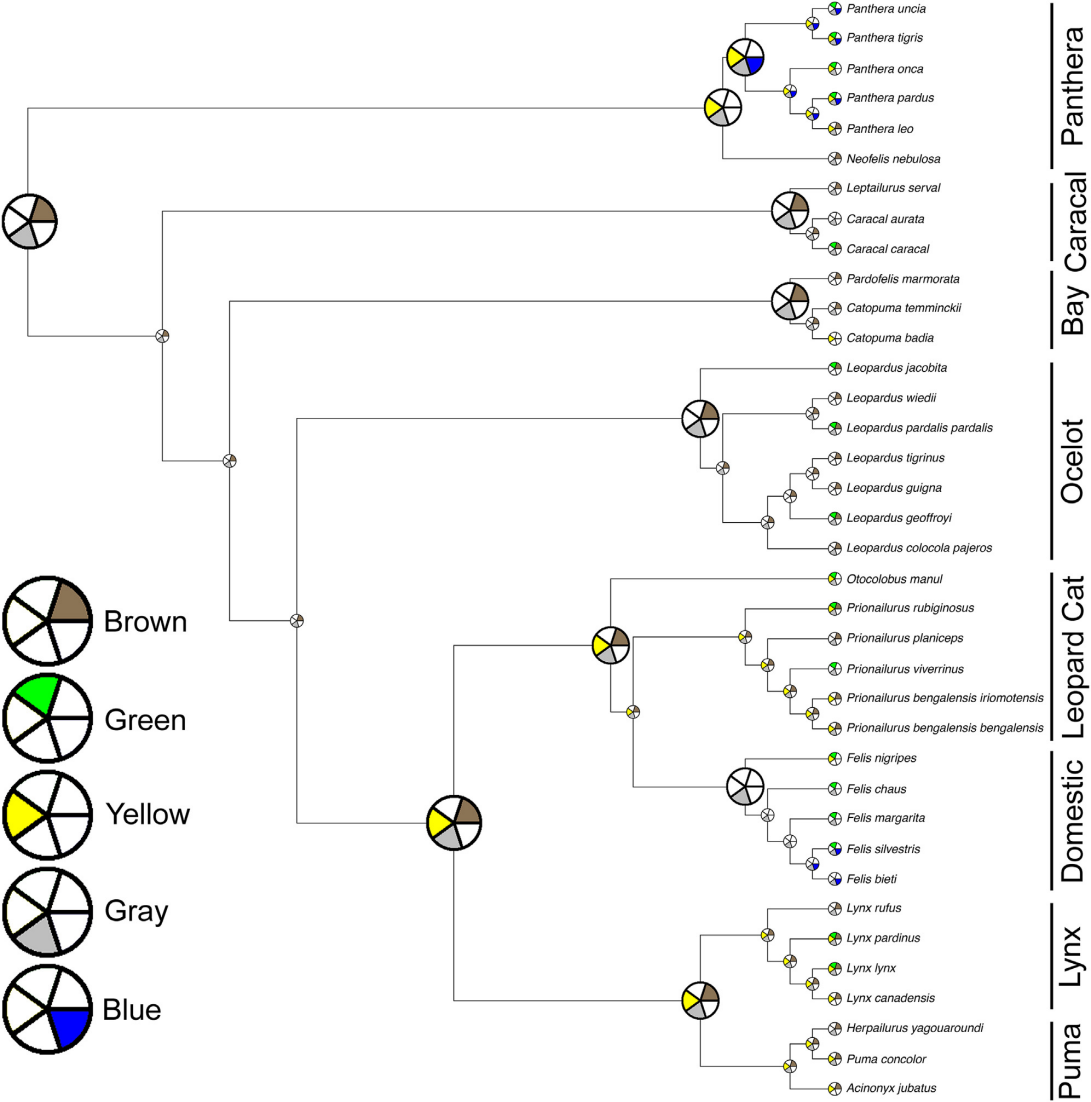
Reconstruction of the ancestral states of all five eye colors for the Felidae The five-wedge pie charts indicate the presence (color) or absence (white) of the various iris colors. Colors were considered present if there was >50% support as determined by maximum likelihood methods (probabilities plotted in Figure S5). Exact branch lengths are not plotted and lineage names are given on the right.

Eye color was reconstructed by subsetting a published ultrametric Carnivora supertree (^19^; see STAR Methods for details). This “main phylogeny” is nevertheless missing nine extant felid taxa, which were added manually (STAR Methods) to generate an expanded “full phylogeny.” The overall ancestral state reconstruction for all of the colors on the main phylogeny is given in Figure 4, with a color considered present if there was greater than 50% support as determined by maximum likelihood methods (i.e., more likely than not). This reconstruction does not substantially differ from the reconstruction for the full phylogeny, with additional species and subspecies added (Figure S4). The only differences are that, for the full phylogeny, there are fewer blue-eyed ancestors on the tree and there is less confidence that the common ancestor of the Ocelot Lineage had gray eyes. Most of the presence/absence information for the colors has a high maximum likelihood of support across the tree (Figure S5). However, a notable exception is green. Under the main tree, taking into account all observed eye colors, the confidence in the presence of green is around 50% for every node, likely due to the wide and seemingly unpatterned distribution of green eyes across taxa. This lack of confidence in the presence of green eyes is ameliorated when either using only the most common eye colors or the full phylogeny, but not when alternate models of trait evolution were tried. In both cases, the models predict the presence of green eyes in the ancestor of the Domestic Cat Lineage with high confidence. The analysis using only the most common eye colors predicts green eyes going back to three more common ancestors. Apart from these few areas, all three analyses are congruent. The reconstructions for all five colors are consistently unclear for the ancestor of the Felidae and *Prionodon linsang*, as well as any of the deeper nodes on the tree. This is unsurprising, given that ancestral state reconstruction becomes more uncertain the farther back one goes.

The ancestor of the Felidae is reconstructed with a high likelihood to have had both brown- and gray-eyed individuals present in its population. This represents two novel changes: having multiple eye colors in the same species (intraspecific eye color variation) and having gray eyes in particular. There is good evidence that this is the only major gain of the gray-eyed trait in the Felidae, although a few taxa subsequently lost it. Brown eyes are also common throughout the tree, but had two major losses, once when the Domestic Cat Lineage diverged 6.2 million years ago and once after the Panthera Lineage diverged 10.8 million years ago.^17^ However, there is uncertainty and discordance between the three analyses about whether the Panthera Lineage ancestor had brown eyes, so this latter brown eye loss may have occurred after the genus *Panthera* split from *Neofelis* approximately 6.5 million years ago. As with gray eyes, there were multiple other species-specific losses, as well as a regaining of brown eyes by *Panthera leo*.

The presence of yellow eyes is predicted to have convergently evolved at least twice in ancestors and multiple more times in individual extant species. The two demonstrated higher than species-level gains of yellow eyes are when the Panthera Lineage split off and when the Lynx, Leopard Cat, Domestic, and Puma Lineages diverged from the rest of the tree (8 million years ago).^17^ Much like the other colors, there were a number of cases of loss and even regaining of yellow eyes.

The presence of green eyes stands apart from the previous three colors because it does not seem to have developed early in the evolution and diversification of felids. Instead, it appears to have evolved at least twelve individual times, most of the time at the species level alone. According to the congruent part of the most common color and full phylogeny analyses, the most significant development of green eyes occurred in the Domestic Cat Lineage when it diverged, only being lost once in that lineage (*Felis bieti*). In fact, that would be the only observed time green eyes were ever lost in the Felidae. The presence of blue eyes has a similar evolutionary distribution to that of green eyes, albeit much more rare, possibly having evolved independently at least twice, according to the main analysis: in the ancestor of the *Panthera* genus and in the ancestor of *Felis silvestris* and *Felis bieti* (also the ancestor of *Felis catus*, not considered in the phylogeny). It should be noted that the most common color and full phylogeny analyses both lead to an alternative prediction that the blue eye color was not present in any of the ancestors and arose in individual tips independently.

### Quantitative ancestral state reconstruction of eye color shades

Beyond assigning eye color to one of the aforementioned five broad color groupings, RGB values from each image were processed using a dimensionality reduction algorithm and examined using a cluster analysis (see STAR Methods for details). This is needed because, while the broad color categories are distinct, it is rare that any given felid eye is homogeneous in color (see Figures 1B, 1C, and 1H for clear examples). This analysis resulted in a quantitative and finely detailed output of the average number of shades for each eye color in each taxon, as well as what the colors of those shades are. All eye colors for all species had between two and four shades (see Figures 1F and 1H for examples of eyes with two and four shades, respectively). Our stringent image selection criteria were shown to provide an accurate and robust assessment of shade coloration, even at very low sample sizes, with little variation due to lighting (Figure S6).

**Figure 5:**
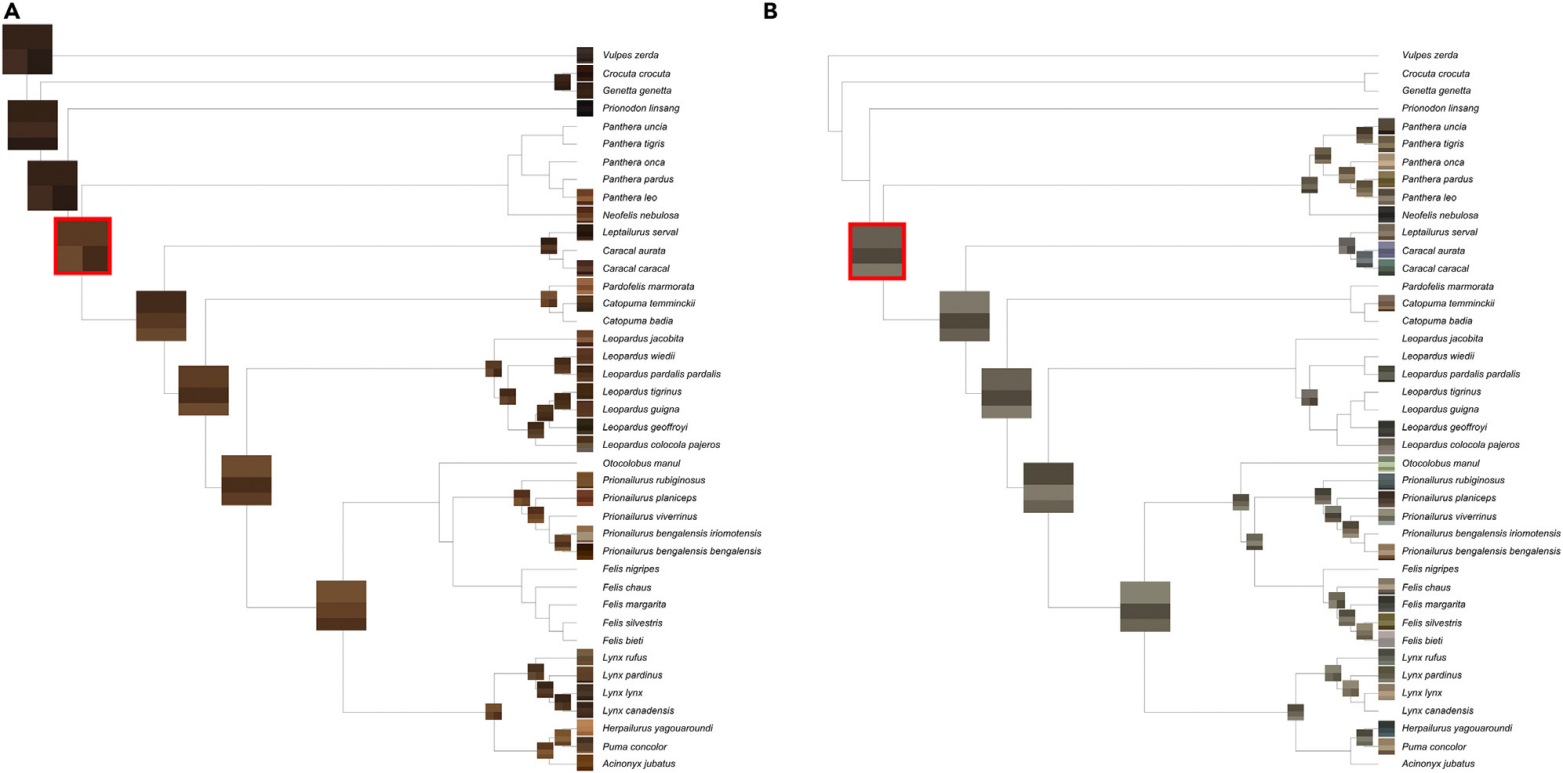
Quantitative reconstruction of the shades of brown and gray eye colors for ancestral felids (A and B) Reconstructions for the shades of brown (A) and gray (B) eyes. The squares at each node are the quantitative reconstructed shades. The proportion of a square that a shade takes up indicates how common that shade is in the data. Exact branch lengths are not plotted. The square highlighted in red is the ancestor of the Felidae.

The reconstruction of the shades of brown eyes, conducted with reference to the presence/absence reconstruction done above, reveals some large-scale evolutionary trends. The eye color of the outgroups (*Vulpes zerda, Crocuta crocuta*, *Genetta genetta*, and *Prionodon linsang*), as well as their close non-felid relatives that were not analyzed in this study, strongly suggests that the ancestor of these groups likely had particularly dark brown eyes, as it is unlikely that similar dark brown eyes would have convergently evolved across closely related and ecologically distinct species. Our study does not have the requisite power to confirm the presence of brown eyes for the common ancestor of the whole tree, but our models do predict that the subsequent ancestors of the outgroups and the Felidae had brown eyes. The predicted darkness is also recapitulated in the reconstruction, including for the common ancestor of the whole tree, if it is assumed to have had brown eyes (Figure 5A). The brown eye colors of all the outgroup ancestors are reconstructed as quite dark, albeit not as dark as the irises of *Prionodon linsang*. The brown eyes of the ancestor of the felids are predicted to have had a lighter coloration, but still with the same shade distribution as the deeper ancestors: the medium shade is the primary shade in the eye, followed by light and dark. After this, the proportion of light, medium, and dark shades changes frequently in the tree. In the data, the medium shade nevertheless remains most likely to be the primary shade (5 for light, 20 for medium, and 7 for dark). The three shades have about an equal distribution of being the secondary shade (10 for light, 11 for medium, and 11 for dark). The overall shade of brown eyes (taking into account dark, medium, and light shades for each) also undergoes substantial changes over the tree. In some lineages, such as the Lynx or Ocelot Lineages, the shade returns to a darker state, as it was before felids branched off. In other lineages, such as the Leopard Cat Lineage, the shade continued to lighten.

A high level of variation in gray eyes is apparent from viewing the types of gray in the data (Figure 5B). Unlike brown, where all of the variety was focused within a relatively narrow region, there are gray colors that span a large spectrum, being closer to brown, green, blue, or yellow for different taxa. The primary gray that was reconstructed for the ancestor of the Felidae (RGB: 118, 112, 100) is closer to brown-gray than pure gray, a trait that continues as the Felidae diversified. Gray colors have close to equal red, green, and blue values, whereas brown colors have much higher red and green values than blue. The ancestral gray having a brownish character is evident by the decreased blue value, compared to the red and green values. The brown content in gray-eyed animals is particularly strong in the Panthera Lineage, eventually nearly becoming fully brown for certain taxa. In the Domestic Cat Lineage, the gray color substantially lightened, losing its brown content and sometimes taking on a slightly higher green content, particularly for *Felis silvestris*. When the genus *Caracal* split from the rest of the Caracal Lineage, its gray changed to have much higher blue and green content (for *Caracal aurata* and *Caracal caracal*, respectively). More blue content in the color of gray eyes is a repeated trait, occurring for *Prionailurus rubiginosus* and *Herpailurus yagouaroundi* as well. For gray, the dark shade is most commonly the primary shade (15 times), closely followed by medium (14 times), then light (9 times). This order is almost reversed for the secondary shades, although it is closer (14, 16, and 8 for light, medium, and dark).

The evolution of yellow eye shades has fewer defined patterns, due to its more recent evolution (Figure S7). The yellow that emerged in the ancestor of the Panthera genus was a brownish yellow, as was the yellow that emerged in the ancestor of the Lynx, Leopard Cat, Domestic, and Puma Lineages. In extant species with the yellow eye color, the brown content in this color is sometimes increased (such as for *Puma concolor*) and sometimes greatly decreased (such as for *Lynx lynx*). For green and blue eye shades, there is even less evidence of a pattern, given the lack of ancestral nodes with the predicted presence of those colors (Figures S8 and S9). An interesting aspect of all three of these colors is that there are a number of species that developed multiple different colors in one eye, rather than (or in addition to) multiple shades. This likely accounts for the majority of the outlier/overlapping points in Figures 3 and S1–S3. An example of this is the blue of *Panthera uncia* (snow leopards). Snow leopard blue eyes routinely have a thin outer band of beige and an inner layer of blue. Our program was easily able to pick this up in its dimensionality reduction, but it should be noted that the reconstruction shades do not have the same level of contrast that the original eyes might have had. This is a result of averaging samples and could be corrected through significantly more data. This issue does not occur for *Panthera pardus*, whose eyes have a similar makeup with a beige outer ring and an inner blue (Figure 1H). Our program is able to output that the beige area is generally about the same size for *Panthera pardus* (primary shade) as for *Panthera uncia* (also primary shade), bypassing what otherwise would have been painstaking morphological measurements.

### Correlation analysis

**Figure 6:**
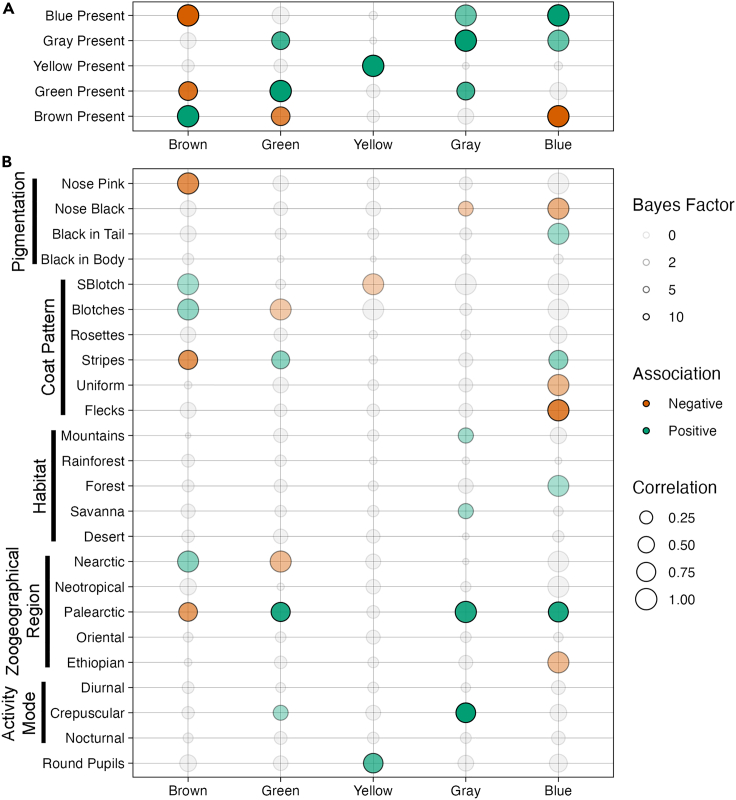
Correlations between the presence of different eye colors and various species traits (A and B) Correlations against the presence of each eye color (A) and against various physical, behavioral, and environmental factors (B). Larger circles correspond to stronger correlations and more opaque circles correspond to more significant correlations. Green circles have a positive correlation, red circles have a negative correlation, and gray circles do not meet the significance threshold (Bayes factor = 2).

The presences of each of the five eye colors were correlated against one another, taking the phylogeny into account (Figure 6A). The correlations for the main phylogeny with all observed eye colors sometimes differ from the results with only the most commonly observed eye colors (Figure S10A) or for the full phylogeny (Figure S10B), but often there is agreement. For all three analyses, a significant positive correlation (log Bayes Factor >2) was identified every time a color was correlated with itself, a positive baseline check of the quality of the method. The main analysis demonstrated a significant negative correlation between the presence of brown eyes and the presence of both green (BF = 8.33, corr = −0.71) and blue eyes (BF = 15.38, corr = −1; indeed, no felid species has both brown and blue eyes). In contrast, the presence of gray eyes is significantly positively correlated with the presence of both green (BF = 7.04, corr = 0.63) and blue eyes (BF = 5.26, corr = 0.98; all blue-eyed taxa also have gray eyes). These correlations for gray, as well as the negative brown-blue correlation, were found for the other two analysis types as well. However, the analysis with just the most common eye colors indicated additional negative correlations between brown eyes and both yellow (BF = 8.87, corr = −0.78) and gray eyes (BF = 7.01, corr = −0.67).

Correlation analysis was also carried out on eye color relative to a variety of other physical traits (e.g., pupil shape, coat color), behavioral traits (e.g., diurnal versus nocturnal activity), and habitat characteristics (Figure 6B). Overall, most environmental and physical factors considered in the analysis showed at least some significant correlations with various eye colors, indicative of the complexity of eye color evolution. Notably, none of the activity modes were correlated with any eye color, except for crepuscularity with gray and green eyes—good evidence that this trait is not particularly important for eye color evolution in felids. Additionally, of the significant correlations, gray eyes were almost always positively associated with other traits. This greatly contrasts with the other eye colors, all of which have closer to a 50/50 distribution of positive and negative associations.

When eye colors were correlated to pupil shape, one significant correlation appeared: the presence of yellow eyes is strongly positively correlated with round pupils (BF = 6.32, corr = 0.80). This correlation was found in both other analyses as well. For all felid taxa that have round pupils, yellow eyes had evolved prior and, even if the taxa subsequently lost yellow eyes, yellow eyes are predicted to have been present in all ancestors that evolved round pupils. Given the above potential negative relationship between brown and yellow eyes, it is also unsurprising that only two taxa evolved round pupils while already having brown eyes (the closely related *Acinonyx jubatus* and *Puma concolor*). It should be noted that one can flip the direction of these correlations to obtain the associations with vertical pupils, given that this is the only other pupil type for felids.

There were some correlations between zoogeographical regions and various eye colors that cannot be otherwise explained by phylogeny, particularly on the ends of the color spectrum: brown eyes are positively associated with the nearctic region (BF = 3.77, corr = 0.97), gray eyes are positively associated with the palearctic region (BF = 8.39, corr = 0.99), and blue eyes are negatively associated with the neotropical region, albeit only in the two alternate analyses (BF = 4.02/4.70, corr = −0.95/-0.96). On the other hand, there were few correlations with habitat.

The traits relating to body pigmentation (coat pattern, black fur present on the tail and the body, and nose color) also revealed few correlations with eye color presence. Having a pink nose is strongly negatively correlated with brown eyes (BF = 6.32, corr = −0.98), the eye color with the highest amount of eumelanin.^20^ Likewise, the eye color with the lowest amount of melanin (blue) is strongly negatively correlated with having a black nose (BF = 4.05, corr = −0.98). Having a coat pattern with blotches or small blotches is highly related to ancestry and these traits are not evenly distributed across the phylogeny.^21^ Because of this, the correlation coefficients between these traits and various eye colors are usually very large (near 1 or -1) and almost entirely explainable by phylogeny, due to coat pattern often being a lineage specific trait. However, two such correlations cannot purely be explained by phylogeny: brown-small blotches (BF = 2.54, corr = 0.97) and green blotches (BF = 2.32, corr = −0.97).

Correlation analysis was also performed for the physical/behavioral/environmental data and the continuous average RGB shade values. This was done because some factors could affect the shades within eye colors, regardless of whether or not they affect the presence of eye color categories as a whole (Figure S11). Many factors are only significantly correlated with one aspect within certain eye colors (out of red, green, and blue shades). Additionally, there does not seem to be much consistency in the effect of various factors across eye colors. Nevertheless, broad trends can be observed. For brown eyes, the shades of red and green, the two colors that make up RGB brown, are what are most often affected by the environmental/behavioral/physical factors, and always in the same direction and magnitude, an expected result and a good check of the method. For green, the green shades are understandably the most affected by environmental/behavioral/physical factors. These results provide additional confidence that the representation of natural colors in a computerized RGB encoding retains biologically relevant information.

## Discussion

Our image-based analysis demonstrates that the family Felidae has a broad range of interspecific and intraspecific variation in eye color, the latter type of variation being conspicuously absent in all the group’s close relatives. Indeed, within-species eye color variation has been thought to be largely absent from wild animals as a whole, except for those species where eye color varies on specific axes, such as age or sex.^6^ While this rule may hold for most groups, the Felidae constitute a notable exception, with over 80% of the taxa surveyed in this study having two or more different eye colors in their populations. The images that make up the dataset were controlled to be all adults, so this cannot be due to a difference between juvenile and adult morphs. Although the sex of animals in the dataset could not be determined, sex alone also cannot account for the variation seen, since 33% of taxa had more than two eye colors in their population. Variation in eye colors to this extent has not been formally described, except in humans and domesticated animals, making the felid system an ideal model to investigate the evolution of eye color.

Our results support the existence of discrete, polymorphic eye color categories for felids, with pixels from brown, green, yellow, gray, and blue eyes separating along biologically meaningful axes, with little overlap between these categories overall. This separation was present for the dataset as a whole, as well as for every species but one. The outlier is *Acinonyx jubatus*, for which there was no statistical support for a distinction between brown and yellow eyes. The fact that this unique overlap between the “yellow” and “brown” categories for *A. jubatus* is not seen in its closest relatives, such as *Puma concolor*, likely indicates that these color categories have recently become more similar in this species. This may be attributed to the lack of genetic diversity in the species and the high level of inbreeding, leading to variation in color being difficult to maintain.^22^

Despite felid eye color being broadly discrete, pigment levels can nevertheless be continuous, and prior studies on color variation suggest that even traits that can be discretized may have continuous variation at a more fine-grain level.^11^ Our finding that there are always at least two shades present within each eye supports this, providing a useful and biologically realistic model for future research into the evolution of eye color variation: treating the overall trait as fully discrete and then treating each category as continuous. This avoids unrealistic transitions between colors (see STAR Methods for an example) in the overall model, while still allowing within-color variation to be analyzed. The quantitative shade analysis also allows eyes with substantial color differences within them to be separated (for examples, see the eyes of *Panthera pardus* in Figure 1H). This variation is retained in our continuous, quantitative analyses (see *P. pardus* in Figure S9). If eye color was treated only as a discrete category, these differences would be ignored, a particularly sizable loss if an eye’s coloration includes colors contrary to the overall color group assigned based on the majority of the eye.

The reconstruction of eye color indicates with high likelihood that the common ancestor population for the Felidae had both brown- and gray-eyed individuals. The presence of brown eyes is not surprising, given that all close relatives of the Felidae have dark brown eyes with no intraspecific variation. However, the presence of gray eyes is likely a family-specific characteristic. Although our analysis has left it unclear whether the ancestor of the Felidae and *Prionodon linsang* included gray eyed individuals, given that all of the close relatives of the Felidae (e.g., genets, hyenas, and so forth) have only brown eyes, it is highly likely that this uncertain ancestor had exclusively brown eyes as well.^17^

The gray eye color is likely an intermediate between all of the other eye colors. Eye color is determined by the amounts of the pigments eumelanin and pheomelanin in the iris.^20^ In a simple view, eyes with more eumelanin are brown, eyes with more pheomelanin are yellow, and eyes with lower levels of pheomelanin and eumelanin are blue or green. Gray eyes contain a moderate amount of both pigments, but not enough of either one to reliably be placed in another color group. This is supported by gray eyes in the data having much higher variability than the other four colors. If a population, such as the relatives of the felids, is homogeneous for dark brown eyes, thus having a high level of eumelanin and little pheomelanin, it would be difficult to suddenly develop blue eyes, given that blue eyes need a very specific balance between the two pigments that is far from the dark brown state. Even a total loss of pigment, as with albinism, could not account for this, because a certain amount of pigment is still needed to have the blue color be visible.^4^

Under this view, once gray eyes evolved in the felid ancestor, it became far easier to transition between eye colors and evolve new ones, resulting in the great diversification seen in the Felidae. It is out of the scope of this study to answer exactly which genetic changes led to this, but this is a question that should prompt future research. A promising starting point is identifying and comparing orthologous sequence data for genes known to affect melanin production in other species, such as OCA2, HERC2, and MC1R, in as many felid species as possible, to attempt to pinpoint felid-specific genetic changes that might affect eye colors.^4^

Further evidence for the evolution of gray eyes being an intermediate form, stemming from a fully brown-eyed population, can be found in the shade reconstruction. The ancestral felid’s gray eyes were not purely gray but were made up of brownish-gray shades. This is only plausible if there is a gradient from brown to gray with no other colors in between and if the gray eyes were formed from a modified brown eye. Furthermore, when examining the quantitative shades of gray across the phylogeny, there are places where other colors were lost, coupled with a shift toward that color by gray. For example, in the genus *Panthera*, when brown was lost, there was a concurrent increase in the amount of brown in the gray-eyed animals. This could be explained by a gradual change of brown eyes becoming more gray, merging the two colors, or by discrete changes, with brown eyes being lost and then gray eyes becoming browner. By the present day, the gray eyes in the *Panthera* have almost crossed back into being brown (for example, *Panthera tigris*). Additionally, there are a number of species for which the content of blue has substantially increased in their gray eyes, such as *Herpailurus yagouaroundi*, *Prionailurus rubiginosus*, and both species in the genus *Caracal*. However, this is never the case for taxa that already have blue eyes, all of which also have gray eyes.

In contrast to *Panthera*, when the Domestic Cat Lineage lost brown eyes, it was coupled with a lightening of the color of gray eyes and likely the evolution of green eyes. In this case, neither of the present colors is close to brown. This represents a second path for the loss of brown eyes: rather than occupying the place of brown eyes in the population by effectively merging brown and gray, the population of the ancestor of the Domestic Cat Lineage shifted the entire eye color scheme. This likely requires a greater number of changes and it is no wonder that such examples of huge eye color scheme shifts are rare. Through comparisons of this nature, the data collected and analyzed in this study can provide important insights into eye color evolution on both small and large scales. It should also be noted that many of the wild species within the Domestic Cat Lineage can breed with the domestic cat (*Felis catus*).^23^^,^^24^ Albeit unlikely, disruption from this form of hybridization is possible, given that many domestic cats have artificially selected eye colors.

Although our method of placing eyes into discrete color categories and then subsequently categorizing their variation in a continuous way is supported by the data, the quantitative reconstruction is subject to a relevant limitation. Since ancestral state reconstruction is applied to each color separately, species with eyes near the border between two color categories are not taken into account in both reconstructions. For example, the ancestor of the Panthera Lineage is predicted to have lost brown eyes and concurrently gained yellow eyes. If yellow eyes directly and gradually evolved from the brown eyes of the ancestor’s ancestor and the new yellow is simply a different shade of brown, then the shades of the yellow eyes of the Panthera Lineage should be taken into account when reconstructing the brown shade of the ancestor’s ancestor. Although this is an unlikely scenario, it is worth noting that it is beyond the scope of our method to account for it unless the discrete categories are ignored, which would lead to a host of far more pervasive and consequential issues.

The correlation results were also revealing. It is unsurprising that brown and blue eyes, at nearly opposite ends of the pigment spectrum, do not frequently coexist in natural populations and are significantly negatively associated. On the other hand, gray eyes, being an intermediate that borders the blue color space, provide an ideal anchor for the rarer blue eyes. The maintenance of blue eyes would be much more likely if blue-eyed individuals mated with blue-eyed or gray-eyed individuals, rather than with brown-eyed individuals. No felids naturally have both brown and blue eyes, so a worthwhile future direction would be to empirically test whether there is segregation in mating preference along eye color lines*.* Mating preferences provide an intriguing possibility for the evolution of eye color differences, given that one hypothesis for the diversity of human eye colors is sexual selection.^3^ Cats are dichromatic and cannot recognize reds and oranges, but they can distinguish other colors.^25^ This range of color sensitivity fits well, given that all the eye colors identified in this study are likely visible to felids and would thus be possible to be sexually selected for. However, even if this was the driving factor behind eye color diversification, it still does not explain the emergence of gray eyes, nor the differences between lineages.

Yellow eyes are also much more likely to coexist with round pupils. Round pupils are a repeated innovation in felids (the ancestral felid had vertical pupils;^26^), so it is notable that they only appeared in ancestors that are predicted to have already developed yellow eyes. Thus, the gain of yellow eyes acts as a prerequisite for the evolution of round pupils. However, our analysis cannot distinguish between correlation and causation. It seems probable that either an aspect of yellow eyes (causation) or an undiscovered lifestyle or physical trait that strongly covaries with yellow eyes (correlation), aligns with the conditions that are ideal for round pupil evolution. While not every species with yellow eyes developed round pupils, the two traits are tightly linked and only *Panthera uncia* lost yellow eyes after evolving round pupils. The reverse of this trend seems to be true for brown eyes since only two groups had brown eyes before evolving round pupils. This makes sense, given the negative association between brown eyes and yellow eyes in the analysis with only the most common eye colors.

The contrasting forces of brown and yellow eyes can be seen in the shade correlations, with yellow eyes lightening the overall red and green shades of a species and brown eyes darkening the overall red, green, and blue shades. If gray eyes developed by decreasing the amount of eumelanin in the eyes, it could be that a second change increased pheomelanin levels, leading to yellow eyes. Then, with a new type of darker pigmented eye, there might have been less of an evolutionary “need” for eyes with lots of eumalanin. This could explain why many species have either brown or yellow eyes, but not both, particularly in lineages that simultaneously gained yellow and lost brown, such as the genus *Panthera*. For such lineages, the concurrent loss of brown and the gain of yellow could also have been achieved if the second change altered the melanin synthesis pathway in brown eyes (as opposed to yellow stemming from gray). This could be achieved by increasing the likelihood that their shared precursor, dopaquinone, becomes pheomelanin, rather than eumelanin.^27^ Either way, the divergent effects of yellow and brown on round pupil evolution fit with these two colors being unlikely to develop together, but not being mutually exclusive.

There were few significant correlations for activity modes, even with the shade data taken into account. This is surprising, given the findings of Passarotto et al.,^15^ which found that darker colored eyes in owls evolved in response to the switch to a nocturnal lifestyle. This is clearly not the case for felids. The ancestral state for felids is nocturnality, but gray eyes (usually lighter than brown eyes) evolved before any taxa made the switch to diurnality.^28^

The fact that there were a number of significant correlations by zoogeographical region is fascinating, given how large each region is. This, coupled with the lack of significant correlations found for most habitats and the uniformity of eye colors across most animals around the world, indicates that the physical environment may play less of a direct role in eye color in felids and possibly mammals as a whole. Future investigations should be done to map eye colors at a population level. This mode of data collection, ideally paired with genomic analyses, would enable full phylogeographic investigations into the evolutionary history of the trait, beyond the broad correlations presented here.

Other traits, such as social system and mode of hunting, are also uninformative, given that the only long-distance (as opposed to short-range ambush) predator felid is the cheetah and the only non-solitary felid is the lion, neither of which shows unique eye color characteristics.^26^^,^^28^ Thus, the specific adaptive benefit of having different eye colors is left as an open question.

It is known that eye color is at least partly tied to coat color in domestic cats and some similar associations do appear in our data, such as having brown eyes being negatively correlated with having a pink nose.^29^ Having a pink nose, an easily measurable partial stand-in for a de-melanated skin color, is unsurprisingly not frequently found in species with brown eyes, which require more melanin. The same logic explains the negative correlation between blue eyes (which need lower melanin) and a black nose (which needs higher melanin). However, for the most part, the color or shade of felid eyes is not related to skin or fur color. This lack of coupling of the two traits, apart from the most and least melanated cases, likely allowed for the independent evolution of gray eyes in the felid ancestor.

All of the evidence presented here supports a larger theory of felid eye color evolution. Through random, novel mutation(s) that decreased the levels of iris eumelanin, a subset of the population of the ancestor of the Felidae developed gray eyes. Once this key innovation occurred, the new standing variation led to rapid diversification. Yellow, green, and blue eye colors evolved repeatedly, albeit not necessarily through the same genetic changes, as felid lineages diverged and groups reached new zoogeographical regions. The presence of these colors and the strength of the shades within them varied tremendously through interactions with different environments and physical characteristics. Tradeoffs between the amounts of pigment in the iris created antagonistic relationships between blue and brown eyes, as well as yellow and brown eyes, making their coexistence in various species less likely. The yellow-brown tradeoff, influenced by a potential increase in pheomelanin, possibly affected the development of round pupils. Iris color diversification represents a defining feature of the Felidae family and the data presented here demonstrates the complexity of the trait.

Eyes, and especially eye colors, have historically been a missed opportunity for evolutionary research. Because they are a rarely preserved element of animal bodies, some have argued that eye color diversification over time can only be studied through ancient DNA.^6^ In contrast to this view, the work presented here demonstrates that eye color can be studied in an evolutionary context, without the need for ancient DNA or even DNA information at all. Through this work, the evolution of eye colors in the Felidae is now much clearer and there are many avenues for more studies, particularly regarding the clearly important evolutionary place that gray eyes occupy. This study provides a starting point for future research into eye color evolution in natural populations, a question that has not had any significant investigations until now and could be easily expanded to other taxa. In addition, the scope of this study could be built upon by adding genetic data to the correlation analysis to work toward answering more functional questions. Beyond this, the method for quantitative color reconstruction presented in this study could be adapted to any color-based analysis, even beyond the iris. This will allow for high precision color reconstructions that were previously impossible.

### Limitations of the study

While ancestral state reconstruction can provide statistical insight into the past, it remains a prediction. This study does not conclusively prove that the ancestor of the Felidae had gray eyes; it strongly predicts that this was the case, given modern data. For taxa where ancient DNA is available from an ancestral population, such as for humans, predictive models can be used to robustly predict eye color information (e.g.,^30^). Thus, one way to be more sure about the eye colors of felid ancestors would be to use the phylogenetic comparative methods presented here in concert with ancient DNA, allowing the predictions made by the models used here to be empirically tested. Of course, a prerequisite to this study would be gaining a firm knowledge of which genes influence modern felid eye color and how their pathways interact, as well as extensive ancient DNA sampling of extinct felids. The felid ancestor existed too long ago for ancient DNA sampling to work in this case, but felids from more recently diverging nodes, such as the ancestor of *Felis bieti* and *Felis lybica*, can still provide a ground truth check for the model’s predictions.

Additionally, despite online databases providing essential support for this work, using such databases limits the amount of data that can be collected on possible confounders. For instance, the age, sex, and provenance of the animals whose photos were used for this study could not be reliably determined. This is especially relevant for rarer species, which are underrepresented in the dataset for this study. While these factors should not have affected the overall conclusions of this study, data was not able to be collected about how eye color varies on these biologically relevant axes.

Lastly, the sampling done for this study is only sufficient to make robust predictions about the Felidae. For a researcher interested in species beyond this family, more data would have to be collected and analyzed using the methods described in this article.

## Resource availability

### Lead contact

Further information and requests for resources should be directed to and will be fulfilled by the lead contact, Julius Tabin (jtabin@g.harvard.edu).

### Materials availability

This study did not generate new unique reagents.

### Data and code availability

• Data: All the raw images in the dataset used for this article are freely available on GitHub at https://github.com/jtabin/Felid-Eyes. Supplemental information and additional data have been deposited at the Dryad Digital Repository and are publicly available as of the date of publication. DOIs are listed in the key resources table.
• Code: All original code has been deposited at Zenodo and is publicly available as of the date of publication. DOIs are listed in the key resources table. The code is also present on the above GitHub page.
• Any additional information required to reanalyze the data reported in this article is available from the lead contact upon request.

## Acknowledgments

We would like to thank Clifford Tabin for methodological and article preparation advice. We want to thank Scott Edwards and his OEB 275R Comparative Genomics seminar, Landen Gozashti, Carolyn Elya, and the members of the Hoekstra Lab at Harvard University for conceptual and methodological assistance. We also would like to thank Liam Revell, Jeremy Beaulieu, Jonathan Schmitt, and especially Michaël Nicolaï for their useful advice on refinements of the methodology. This work was supported in part by a graduate stipend from the Department of Organismic and Evolutionary Biology at Harvard University and published by a grant from the Wetmore Colles Fund.

## Author contributions

Conceptualization, J.A.T.; data collection and preparation, J.A.T. and K.A.C.; data analysis, J.A.T.; writing – original draft, J.A.T.; writing – review and editing, J.A.T. and K.A.C.; supervision, J.A.T.

## Declaration of interests

The authors declare no competing interests.

## STAR★Methods

### Key resources table

**Table:** 

REAGENT or RESOURCE	SOURCE	IDENTIFIER
**Deposited data**		

Raw and analyzed data	This paper	https://doi.org/10.5061/dryad.s4mw6m9b0
Felid Images	This paper	https://github.com/jtabin/Felid-Eyes
Phylogeny	Nyakatura and Bininda-Emonds^19^	https://doi.org/10.1186/1741-7007-10-12

**Software and algorithms**		

iNaturalist	iNaturalist^31^	https://www.inaturalist.org/
Python (version 3.8.8)	Van Rossum and Drake^32^	https://www.python.org/downloads/
Color Names	Aerne^33^	https://github.com/meodai/color-names
Color Namer	Cooper^34^	https://github.com/robertcoopercode/color-namer
BayesTraits (version 3.0.5)	Pagel et al.^35^	https://www.evolution.reading.ac.uk/BayesTraitsV3.0.5/BayesTraitsV3.0.5.html
R (version 4.2.1)	R Core Team^36^	https://cran.r-project.org/bin/macosx/

### Experimental model and study participant details

All images used in this study were taken from pre-existing digital sources, all experiments were purely computational, and no physical subjects, living or dead, were used in this work. Images were controlled to all be of healthy adult individuals, ideally residing in the wild. Sex could not be reliably determined for every individual, but this should not substantially limit the generalization of the results. For further discussion about the possible influence of sex on the results of this study, see the discussion and the limitations of the study sections. The scientific names, common names, and image sample sizes for each of the 56 taxa considered in this study are present in Table S2.

### Method details

#### Data set

In order to sample all felid species, we took advantage of public databases. Images of individuals from 40 extant felid species (all but *Felis catus*, excluded due to the artificial selection on eye color in domesticated cats by humans), as well as 12 identifiable subspecies and four outgroups (banded linsang, *Prionodon linsang*; spotted hyena, *Crocuta crocuta*; common genet, *Genetta genetta*; and fennec fox, *Vulpes zerda*), were found using Google Images and iNaturalist using both the scientific name and the common name for each species as search terms.^31^ This approach, taking advantage of the enormous resource of publicly available images, allows access to a much larger data set than in the published scientific literature or than would be possible to obtain *de novo* for this study. Public image-based methods for character state classification have been used previously, such as in a phylogenetic analysis of felid coat patterns^21^ and a catalog of iris color variation in the white-browed scrubwren.^37^ However, this approach does require implementing strong criteria for selecting images.

Criteria used to choose images included selecting images where the animal was facing towards the camera, at least one eye was unobstructed, the animal was a non-senescent adult, and the eye was not in direct light (causing glare) or completely in shadow (causing unwanted darkening). The taxonomic identity of the animal in each selected image was verified through images present in the literature, as well as the “research grade” section of iNaturalist. When possible, we collected five images per taxon, although some rarer taxa had fewer than five acceptable images available. In addition, some species with a large number of eye colors needed more than five images to capture their variation, determined by quantitative methods discussed below. Each of the 56 taxa and the number of images used are given in Table S2.

Once the images were selected, they were manually edited using MacOS Preview. This editing process involved choosing the “better” of the two eyes for each image (i.e. the one that is most visible and with the least glare and shadow). Then, the section of the iris for that eye without obstruction, such as glare, shadow, or fur, was cropped out. An example of this is given in Figure S12. The strict selection criteria and image editing eliminated the need to color correct the images, a process that can introduce additional subjectivity; the consistency of the data can be seen in the lack of variation between eyes identified as the same color (Figure S6). This process resulted in a data set of 290 cropped, standardized, irises. These images, along with the original photos, can be found in the supplemental information.

#### Eye color identification

To impartially identify the eye color(s) present in each felid population, the data set images were loaded by species into Python (version 3.8.8) using the Python Imaging Library (PIL).^32^^,^^38^ For each image, the red, green, and blue (RGB) values for each of its pixels were extracted. Then, they were averaged and the associated hex color code for the average R, G, and B values was printed. The color associated with this code was identified using curated and open source color identification programs.^33^^,^^34^ There is no universally agreed upon list of colors, since exact naming conventions differ on an individual and cultural basis, but these programs offer a workable solution, consisting of tens of thousands of colors names derived from published, corporate, and governmental sources. This data allowed the color of each eye in the data set to be impartially assigned, removing a great deal of the bias inherent in a researcher subjectively deciding the color of each iris.

Eye colors were assigned on this basis to one of five fundamental color groups: brown, green (including hazel), yellow (including beige), gray, and blue. The possible color groups were determined before observation of the data based on basic color categories established in the literature: white, black, red, green, yellow, blue, brown, purple, pink, orange, and gray.^39^ Of course, not all of the eleven categories ended up being represented by any irises; no irises were observed to be white, black, red, purple, pink, or orange.

As an example of this method, if an iris’s color had the RGB values R: 114, G: 160, B: 193, this would correspond to the hex code #72A0C1. This hex code, when put into the color identification programs, results in the identification “Air Superiority Blue”, derived from the British Royal Air Force’s official flag specifications.^33^^,^^34^ Based on the identification, this iris would be added to the “blue” color group, bypassing a researcher having to choose the color themself. If a color’s name did not already contain one of the eleven aforementioned color categories, the name was searched for in the Inter-Society Color Council-National Bureau of Standards (ISCC–NBS) System of Color Designation.^40^ For instance, the color with RGB values R: 37, G: 29, B: 14 corresponds to hex code #251D0E, identified as “Burnt Coffee” by the color identification programs. The ISCC–NBS descriptor for this color is “moderate brown”, so the color would be added to the “brown” group. All colors were able to be placed directly from their color name or their ISCC–NBS descriptor and, for colors with both a color category in the name and an ISCC–NBS descriptor, there were no instances in which the two conflicted.

While color itself lies on a spectrum, splitting the colors into discrete fundamental groups is the most tractable approach to analyze eye color in a biologically reasonable way. If every eye color was instead taken together on one spectrum and analyzed as a continuous trait, the results would be highly unrealistic. As an example, if there were two sister taxa, one with blue eyes (R: 0, G: 0, B: 139) and one with brown eyes (R: 150, G: 75, B: 0), a continuous reconstruction would assign the ancestor the intermediate eye color in the color space: R: 75, G: 37, B: 69. However, this color is firmly within the “purple” category. It is highly unlikely that a recent ancestor of two taxa with blue and brown eyes had purple eyes, rather than blue eyes, brown eyes, or both, which would be the result if blue and brown were considered as separate categories. Indeed, one would run into the same issue if categories were removed at an earlier stage and each taxon was only considered to have one eye color, determined by averaging all irises. A taxon with blue and brown eyes would again be said to have purple eyes, a color which none of the members of that taxon have. The data being separated into color groups is the most realistic way to investigate this trait, preventing the loss of variation present in the natural populations and simultaneously creating impossible analyses. The lines between color categories are not always clear to an observer (e.g. grayish-blues and bluish-grays can look alike) and, no matter how they are defined, they may still be arbitrary. Nevertheless, this is why we used color identification programs, impartially defining the lines to make the analysis possible.

To ensure no data was missed due to low sample size, the first 500 Google Images, as well as all the “research grade” images on iNaturalist, were manually viewed for each species, while referring back to already analyzed data and periodically checked with the color identification programs.^33^^,^^34^ Any missed colors were added to the data set. This method nonetheless has a small, but non-zero, chance to miss rare eye colors that are present in species. However, overall, it provides a robust and repeatable way to identify the general iris colors present in animals.

In addition, if, for a given species, one, two, or three eye colors were greatly predominant in the available data online (i.e. the first 500 Google Images, as well as all the “research grade” images on iNaturalist), they were defined as being the most common eye color(s). For three colors to be considered the most common, each color had to be present for >26.6% of the images. For two colors, each had to be present for >40% of the images. If neither of these conditions were met, the eye color present in the highest percentage of the images was the single most common eye color. The cutoff of 20% was used for four colors, but no species had four colors that met that threshold. With this assessment, the phylogenetic analysis below could be carried out with all recorded eye colors, as well as using only the most common eye colors, thereby assuring that rare eye colors did not skew the results.

#### Color polymorphism assessment

Although placing the eyes in the data set into discrete color groups is useful for downstream analyses, we also wanted to make sure a polymorphic assessment of the iris color trait reflects the reality of the trait. To do this, we performed a principal component analysis (PCA) on the R, G, and B values of every pixel of every iris in the data set, using the package scikit-learn (version 1.2.0) in Python and the built-in stats package in R (version 4.2.1).^36^^,^^41^ The utility of PCA for color polymorphism assessment has been demonstrated before.^10^ By averaging the pixels, irrespective of color group, in twenty equally spaced bins along each of the first three principal components (PCs), we were able to get a sense of what aspect of the color variation each PC was capturing. Then, we fit a linear mixed model for eye color on each of the PCs using the R package lme4 (version 1.1.34), including the species and individual the pixels were coming from as nested random effects.^42^

This method allowed us to compare the effect of assigned iris color along each principal component axis using Satterthwaite’s t-test with Bonferroni correction. A significant effect of color group for a given PC in the linear mixed model would indicate that the color category assigned according to the methods above is meaningfully predictive of a pixel’s value along that PC. As an example, if the real irises are not adequately represented by the discrete color categories proposed here (e.g. brown eyes are all brownish-gray and gray eyes are all grayish-brown, so the categories significantly overlap), then there should not be a significant effect of assigned eye color for a PC that separates pixels by color (e.g. a PC that separates gray and brown). Of course, due to the nature of converting not fully standardized photographs into pixel data, there are many individual pixels that are outliers within a given eye—for instance, a brown-colored pixel might show up in an eye categorized as “blue” because of a fleck of dust in the cat’s eye or some irregular pigmentation—but, unless these outliers are numerous (thus making them not outliers), they should not affect this analysis.

If a significant effect of a color group was found for a PC, the PC values for all categories were compared to one another using a post-hoc Tukey HSD test with Bonferroni correction from the package emmeans (version 1.8.8), in order to distinguish which groups in particular significantly differ for that PC.^43^ Although this analysis was able to determine which color groups adequately reflect the true trait distributions and are meaningful overall, this does not necessarily mean that a polymorphic view of eye color is appropriate for all species. To address this, since PC2 was demonstrated to be the axis that separates pixels by relevant colors, the pixels in each color group for each species were compared along PC2 using a Kruskal–Wallis test with Bonferroni correction to determine whether there was a significant effect of color group at all. If there was, a two-sided pairwise Mann-Whitney-Wilcoxon test with Bonferroni correction was used to compare each group to one another. In this way, we were able to determine the biological appropriateness of using discrete color categories to analyze felid iris color.

#### Shade measurements within each color group

Although averaging the pixels within each iris was sufficient to categorize the colors present for each felid taxon, not every felid iris has homogeneous pigmentation. For example, some colors in some taxa are subject to central heterochromia with a darker pigment near the pupil and a lighter pigment in the periphery (Figures 1B and 1H) or the reverse (Figure 1C). Thus, we calculated corresponding “shade” values for each color group in each species. To do this, the images were sorted into their color groups for each species. For each group, RGB values for each pixel in each image were again extracted, resulting in a three-dimensional data set. This was reduced to two dimensions using Uniform Manifold Approximation and Projection (UMAP), a method selected for its preservation of local structure, important for potential fine shade differences.^44^ The UMAP projection for each image was then analyzed using k-means clustering through scikit-learn.^41^ The number of clusters (k), indicating the number of distinct shades of color in the iris of each animal, was determined using elbow plots.

After this was done for all images in the group, the k values were averaged and each image was clustered using the average k value, rounded to the nearest integer. This was done to standardize within groups, avoid confounders based on lower quality images, and allow for comparative analysis. After this, the average RGB values for each cluster for each image were calculated. Then, the clusters were matched up based on similarity. To do this, one image from the group had its clusters labeled in order (if there were three clusters, they would be 0, 1, and 2). Then, another image from the group would have the distances in 3D space between each of its clusters compared to each of the labeled clusters. The optimal arrangement of clusters was found by calculating the sum of squared errors for every possible combination of clusters and taking the minimum. Then, the clusters were merged. This method was repeated for every image in the group. Doing this for every color of every species resulted in an output with the number of shades within the iris for each color in each species, as well as an average of each different shade across the data. Throughout this process, images were not resized so as to allow higher quality images with more pixels to contribute a greater amount to the average. This was done to ensure any blurring from lower quality images did not obscure the true shade variety in each eye.

The final, combined clusters were ranked by how prevalent they were within the eyes, calculated by the number of pixels in each group, and the groups for each shade were categorized as “dark”, “medium”, or “light”. To do this, if there were three general clusters for a color of a species, the distance from black (RGB: 0,0,0) in 3D space for each of the cluster average RGB values was computed and then they were assigned to be “dark”, “medium”, or “light” based on increasing distance from black in the color space. For species with two shades in their eyes of a certain color, the cluster average RGB values were compared, again using distance, to the averages of the three-shade eye “dark”, “medium”, and “light” values. They would be assigned the label that they were closest to. The remaining space was filled: if “dark” or “light” was empty, the “medium” value was duplicated; if “medium” was empty, the “dark” and “light” values were averaged. This method allows two-shade eyes to be compared to three-shade eyes without losing vital information. For species with one shade of a color in their eyes (of which there ended up being none in the data set), its average RGB values were assigned to “dark”, “medium”, and “light”. Lastly, eyes with four shades had to combine the two most similar shades together in order to make them comparable to the rest of the data set. The importance of this pipeline is to create a data set that can be compared in a standardized way. The information about which shades are most represented was also collected and saved. This data can be found in the supplemental information.

To ensure these results were accurately assessing eye color, the RGB values for each shade within each species were compared with increasing numbers of images from the data set (for examples, see Figure S6). If the RGB values leveled off as sample size increased, that would indicate that the sample is representative of the “true” shades. If there were major fluctuations, that would indicate that the sample size is not high enough to overcome variations in lighting conditions. In this way, the sample sizes for each color present in each taxon were confirmed to be sufficient as their RGB values leveled off.

#### Phylogeny

The phylogeny used for this work was a subset from the Carnivora supertree from Nyakatura and Bininda-Emonds.^19^ This ultrametric phylogeny takes into account 188 literature and gene trees and includes members of all eight Felidae lineages. More recent phylogenies are largely congruent, differing mainly in the placement of the Bay Cat Lineage and the Pallas’s cat (*Otocolobus manul*), partly due to differences in Y chromosome evolutionary evidence compared to other lines of evidence.^45^ Alternate placements were tested and were found to not produce a significant difference in results, making these discrepancies irrelevant to this study.

This Carnivora supertree tree is missing nine of the extant felid groups for which data was collected. Thus, a second tree (termed the “full” tree) was created with the missing species being added manually according to their placements on a Felidae specific tree from Johnson et al.^17^ and/or the more recent tree from Li et al.^45^ The subspecies added were defined according to the most recent identification based on Kitchener et al.^46^ and Liu et al.^47^ Subspecies were added as a polytomy next to the previously defined species on the tree. Since divergence data was unavailable for some of the species and subspecies, the additions were made with branch lengths equal to the nearest resolved neighboring branch, a severe overestimation of the divergence between groups.

It is important to note that this method of manually adding taxa to a tree is flawed without proper sequence data and certainly should not be relied upon for ancestral state predictions or to make broad claims, as there is no guarantee that any addition reflects true divergence. However, this tree was created purely to provide some insight into local areas of the tree at the species level (e.g. what was the eye color of the ancestral tiger?). Even still, these predictions must be understood as far more uncertain than analyses with the original supertree with more limited taxa. The full tree with all the eye colors present for each species is shown in Figure 2. The main tree created only considering the most common eye colors is presented in Figures S4A–S4E.

#### General color reconstruction

To begin the process of ancestral state reconstruction, the phylogenetic trees were read into R using the package ape (version 5.6-2).^48^ A table of taxa, and the colors represented for each, was loaded in and scored with 0/1 for absence/presence. The same table with just the most common eye colors was also loaded in.

The optimal model of trait evolution was then determined for each of the five eye colors independently across the tree. This was done using an Akaike information criterion (AIC) analysis done on the results of 14 different models run using the R package phytools (version 1.2-0).^49^ Six of the models were run using equal/symmetric rates (ER): a continuous-time Markov (Mk) model using fitMK(); an Mk model with edge rates assumed to have been sampled randomly from a Γ distribution using fitgammaMk(); a hidden rates model with four hidden states using fitHRM(); a hidden rates model with six hidden states using fitHRM(); a hidden rates model with a possible hidden state when a color is present, but not when it is absent, using fitHRM(); and a hidden rates model with a possible hidden state when a color is absent, but not when it is present, using fitHRM(). Another six of the models were identical, except they were run using asymmetric rates (ARD). The final two used an Mk model, but assumed that a color cannot be lost after it is gained or that it cannot be gained after it is lost, respectively. This process was done for the data of all the observed eye colors, as well as for the data for the most common eye colors and for the full phylogeny, with the AIC output and weights for the ER and ARD models given in Table S3. The model with the lowest AIC value, indicating the best explanation of the data given the number of parameters, was used for subsequent analyses.

In the main analysis, the best models for trait evolution were found to be: an All Rates Different extended Mk model for brown and blue eyes, meaning that the transition from absence to presence and presence to absence had different rates; an Equal Rates Mk model for green eyes, meaning that the transition rates from absence to presence and presence to absence were identical; an Equal Rates model with a hidden rate when the color is absent for yellow eyes; and an All Rates Different model with a hidden rate when the color is absent for gray eyes. These best models were also rerun in corHMM (version 2.8) and no differences between the results were found.^50^

Although the presence/absence of each eye color were analyzed on their own, the colors are likely not fully independent. Therefore, they were also analyzed together as a polymorphic trait using stochastic mapping through fitpolyMk() in phytools. Since there were far too many states (2^5^-1), including high parameter complexity, for adequate interpretation as a polymorphic character and the two analyses generally aligned (data not shown), the independent model was used. A color was said to be present at any given node (Figure 4) if the marginal maximum likelihood ancestral state reconstruction for that color was greater than 50%, indicating more support for presence than absence.

#### Quantitative color reconstruction

After data was collected on the eye colors present for every node on the tree, more specific reconstructions were possible. For each node, a new tree was created for each eye color present at that node. Each of these subset trees included every descendant of that node that shared each eye color with it, except for those where the color was lost and then re-arose independently. For example, an ancestral node that was determined to have green eyes and brown eyes present would have one tree with all its continuous, green-eyed descendants and another tree with all its continuous, brown-eyed descendants. A diagram of this method is given in Figure S13. This method was done to most accurately reconstruct along plausible evolutionary pathways. If one wants to predict the eye shade of a specific color for a specific node, one should omit taxa that either have lost that eye color (since their present condition cannot communicate any relevant information about the shade of that color for their ancestor), as well as taxa that have lost that eye color and then regained it (since it is unknown whether their present condition is at all related to the shade of that color for their ancestor).

After the trees were created, the specific colors were reconstructed using maximum likelihood methods with the function fastAnc() from phytools.^49^ This was done independently for the red, green, and blue values for each of the data sets collected for the light, medium, and dark shades. Since RGB values can only be from 0-255, it was heartening that the 95% confidence intervals for the quantitative reconstructions were almost always well within the realistic range, lending considerable support to the reconstructions. Large confidence intervals are a known limitation of continuous trait likelihood reconstructions, so one should not understand the reconstructions to always communicate the exact eye shades of the felid ancestors, but they are useful in comparison to one another to illuminate larger trends.

Beyond reconstructing the colors themselves, corHMM’s rayDISC() was used to reconstruct the number of shades within each eye color for each node, using the shade representation data as a discrete, multistate trait.^50^ This was also done for the primary and secondary shades within each eye. Put together, these methods allow for a high resolution understanding of the iris color of ancestral felids. For each ancestral felid population, we are able to know: which color eyes were present (out of brown, green, yellow, gray, and blue), how many different shades they had in their eyes for each color, which shades were more or less common, and approximately what those shades would have been.

#### Environmental/behavioral/physical trait data

Data on pupil shape was obtained from Banks et al. and data on activity by time of day and primary habitat(s) was obtained from the University of Michigan Animal Diversity Web.^26^^,^^28^ Data on zoogeographical regions were based on Johnson et al.^17^ and data on coat patterns were based on Werdelin and Olsson.^21^ Nose color data (pink or black) and whether or not any black was present in the coat or tail were determined manually from observation of images.

For correlation comparisons, each multistate trait was converted into a set of binary traits. Pupil shape was scored with 0 for vertical/subcircular pupils and 1 for round pupils. Likewise, whether black coloration is present in the coat and whether black coloration is present in the tail were scored with a 0 for absence and a 1 for presence. Activity was split into three traits, each corresponding to an activity lifestyle: nocturnal, crepuscular, and diurnal. Then, for each felid taxon, each trait was scored as present or absent using 1 or 0, respectively. This was especially useful, given that some taxa fall into multiple categories. The same was done for historical zoogeographical region (nearctic [North America], neotropical [South America], palearctic [Europe and North Asia], oriental [South Asia], ethiopian [Africa]), primary habitat (mountains, rainforest, forest, savanna, desert), and coat pattern (flecks, uniform, stripes, rosettes, blotches, sblotch [small blotches]). This method was also used for pink and black nose colors because some felid noses contain both colors and some species have both of the colors represented in their populations; in both cases, both colors would be marked as present.

#### Correlation analysis

Apart from reconstructing ancestral states, different correlations were performed in order to investigate the possible evolutionary interactions related to eye color variation. The environmental/physical trait data, along with the presence/absence data for each eye color, was analyzed with a maximum likelihood approach using BayesTraits (version 3.0.5), made accessible in R through the package btw (version 2.0).^35^^,^^51^ This was done by building two models, one where the evolution of two binary traits is independent and one where their evolution is dependent on one another (i.e. where the rate of change in one trait is influenced by the state of the other trait). Then, the models were evaluated using a calculated log Bayes Factor, with a log Bayes Factor over 2 indicating positive evidence for the dependent model. Given the stochasticity of these models, the model comparisons were done 100 times and the calculated log Bayes Factors were averaged, ensuring robust and reproducible results. This process was done by comparing the presence of each eye color to all others, as well as the environmental/behavioral/physical data to the presence of each eye color, the average shade of the RGB values in each eye color, and the average shade of the RGB values in all eye colors overall. This latter average was computed for all taxa by dropping NA values in the averages. To transform the average values into discrete traits, each value was categorized using Jenks natural breaks optimization, performed through the getJenksBreaks() command in the package BAMMtools (version 2.1.10).^52^ Finally, tetrachoric correlation coefficients were calculated using the tetrachoric() command in the package psych (version 2.2.9), to indicate the direction of each association.^53^ For the shade correlations, a positive association indicates that the trait is associated with lighter shades.

### Quantification and statistical analysis

Statistical tests, software used, sample sizes, and corrections for multiple comparisons are given in the text and figure legends. In figures, when asterisks were used for significance, they were as follows, after multiple testing correction: ns: p > 0.05; ∗: 0.05 > p > 0.01; ∗∗: 0.01 > p > 0.001; ∗∗∗: 0.001 > p > 0.0001; ∗∗∗∗: 0.0001 > p.

## Supplemental information

Document S1. Figures S1–S13

Table S1. Results of statistical tests on the principal components of the RGB values for all pixels in the dataset, related to Figure 3

Table S2. The number of images per felid species in the overall dataset, related to STAR Methods

Table S3. Results of phylogenetic model comparisons, related to STAR Methods
