# Supplementary material for: Evolutionary insights into Felidae iris color through ancestral state reconstruction
Source: iScience. 2024 Sep 13;27(10):110903. doi: 10.1016/j.isci.2024.110903 (PMC11465125; doi:10.1016/j.isci.2024.110903)
Supplement: Document S1. Figures S1–S13 [file mmc1.pdf]

iScience, Volume *27*

Supplemental information

Evolutionary insights into Felidae iris color through ancestral state reconstruction

Julius A. Tabin and Katherine A. Chiasson

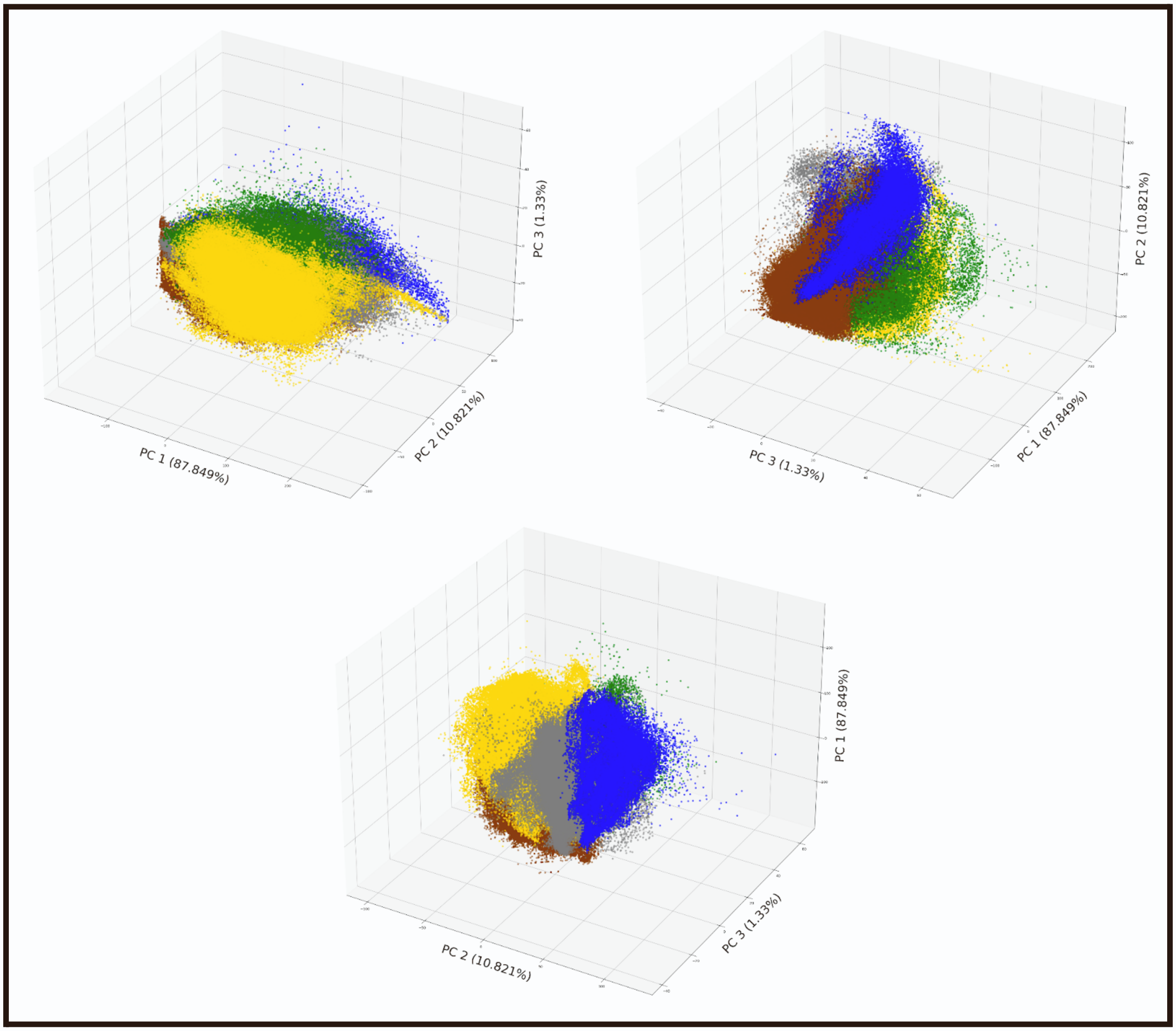

Figure S1: Three angles of a 3D plot of all pixels in the data set along PCs 1-3, related to Figure 3. The three graphs all present the same data, the only difference being the orientation of the axes. Each point is colored by the color category for the respective eye the pixel came from (brown: brown eyes, green: green eyes, yellow: yellow eyes, gray: gray eyes, blue: blue eyes). General separation of color categories can be seen.

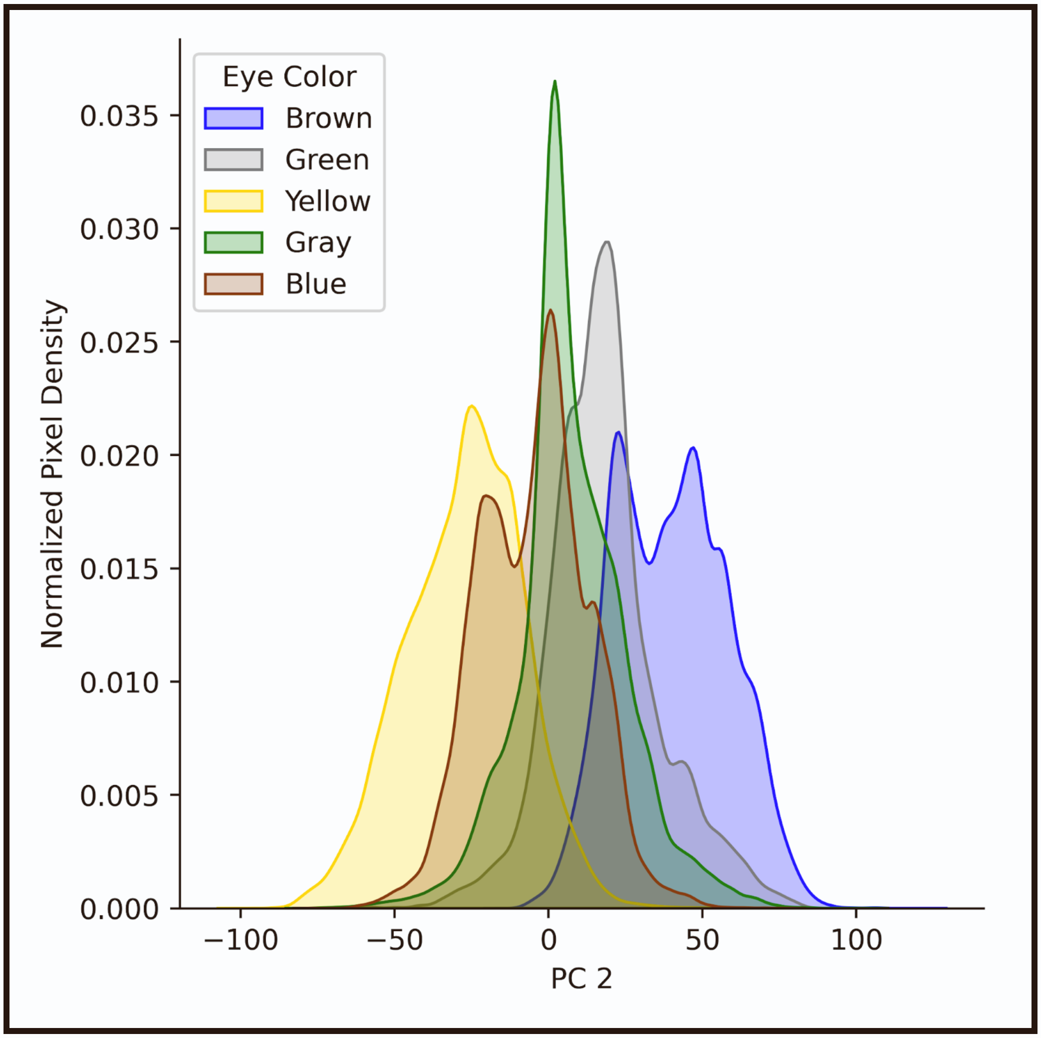

Figure S2: Density of all pixels in the data set along PC 2, separated by eye color, related to Figure 3. The density for each color category was normalized based on the overall pixel number, preventing more common eye colors from overwhelming the plot.

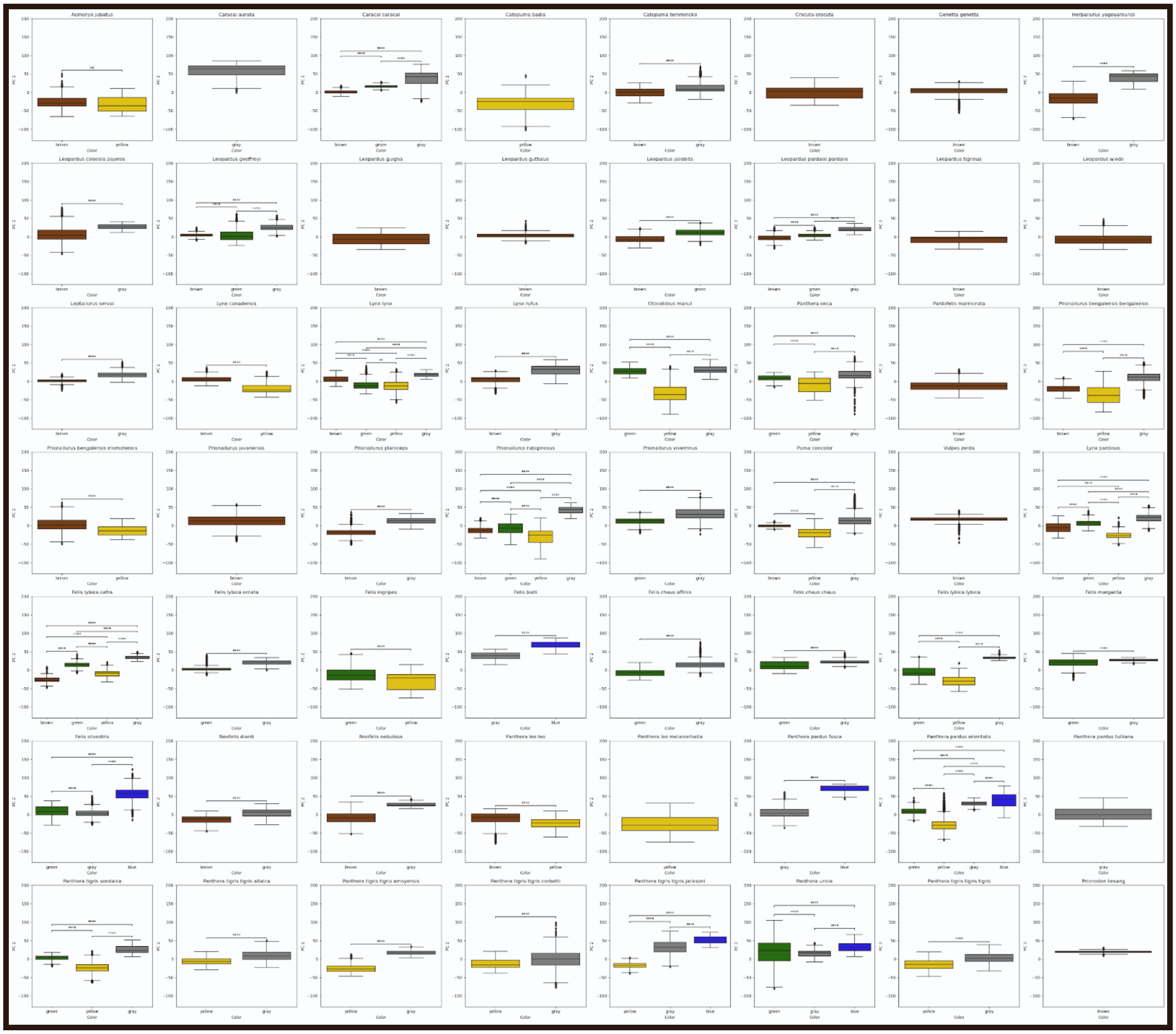

Figure S3: Distribution of pixels for each species for each of the color categories for PC 2, related to Figure 3. Significances calculated using pairwise Mann-Whitney-Wilcoxon tests, two-sided with Bonferroni correction. ns: $p > 0.05$; *: $0.05 > p > 0.01$; **: $0.01 > p > 0.001$; ***: $0.001 > p > 0.0001$; ****: $0.0001 > p$. Data are represented as IQR +/- 1.5*IQR.

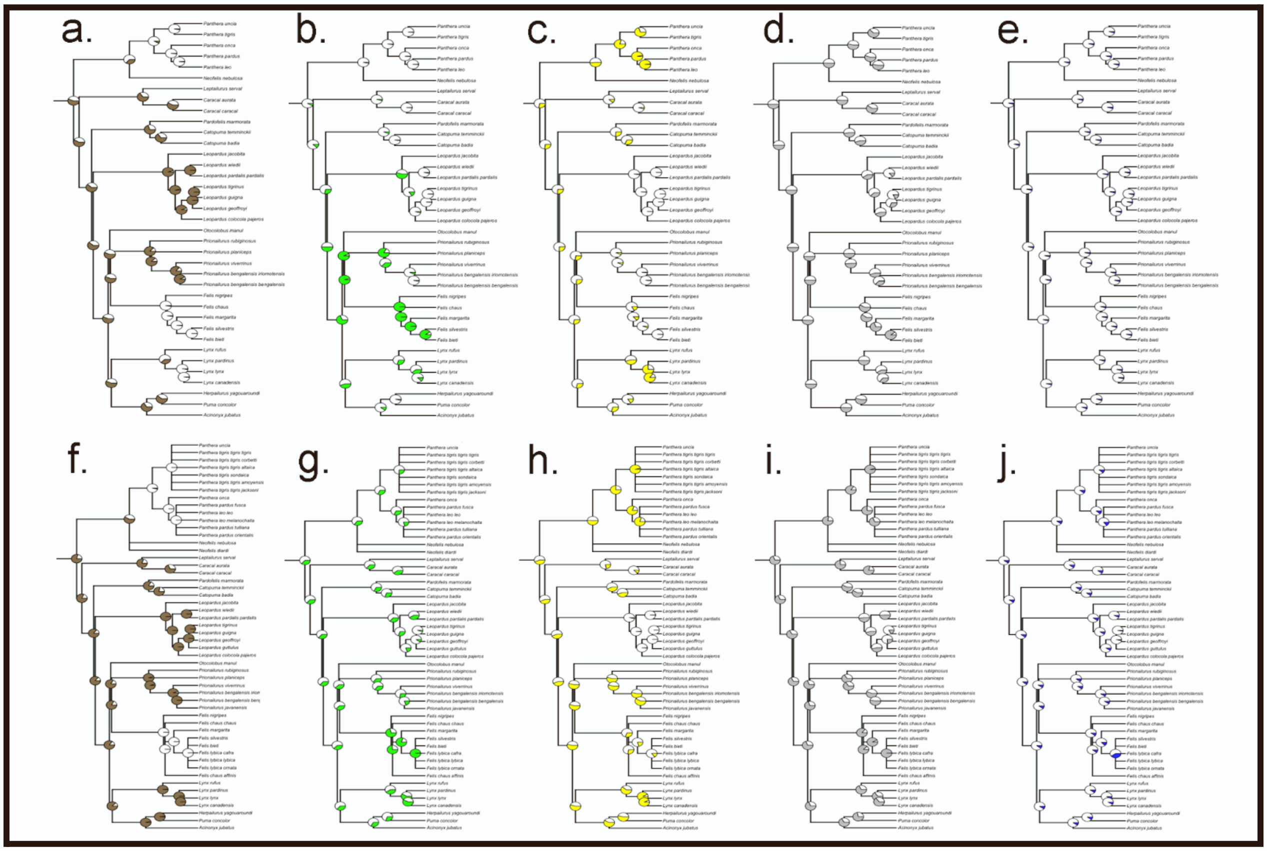

Figure S4: Maximum likelihood probabilities for the ancestral states of brown, green, yellow, gray, and blue eye colors when only the most common eye colors were considered (a-e) or all of the subspecies were added to the tree (f-j), related to Figure 4. The amount of color in the node pie charts represents the support for that color being present at that node. Exact branch lengths are not plotted.

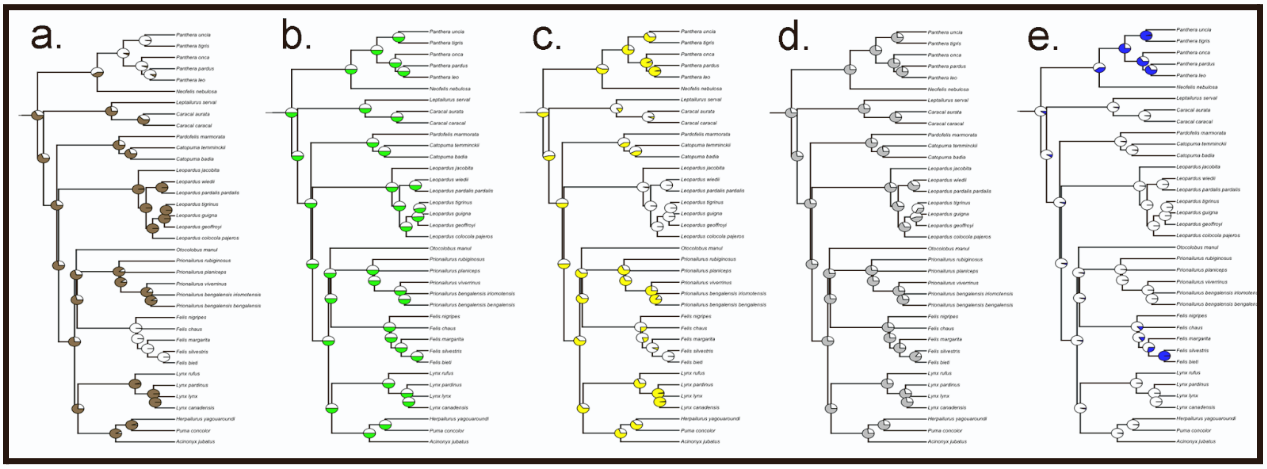

Figure S5: Maximum likelihood probabilities for the ancestral states of brown (a), green (b), yellow (c), gray (d), and blue (e) eye colors, related to Figure 4. The amount of color in the node pie charts represents the support for that color being present at that node. Exact branch lengths are not plotted.

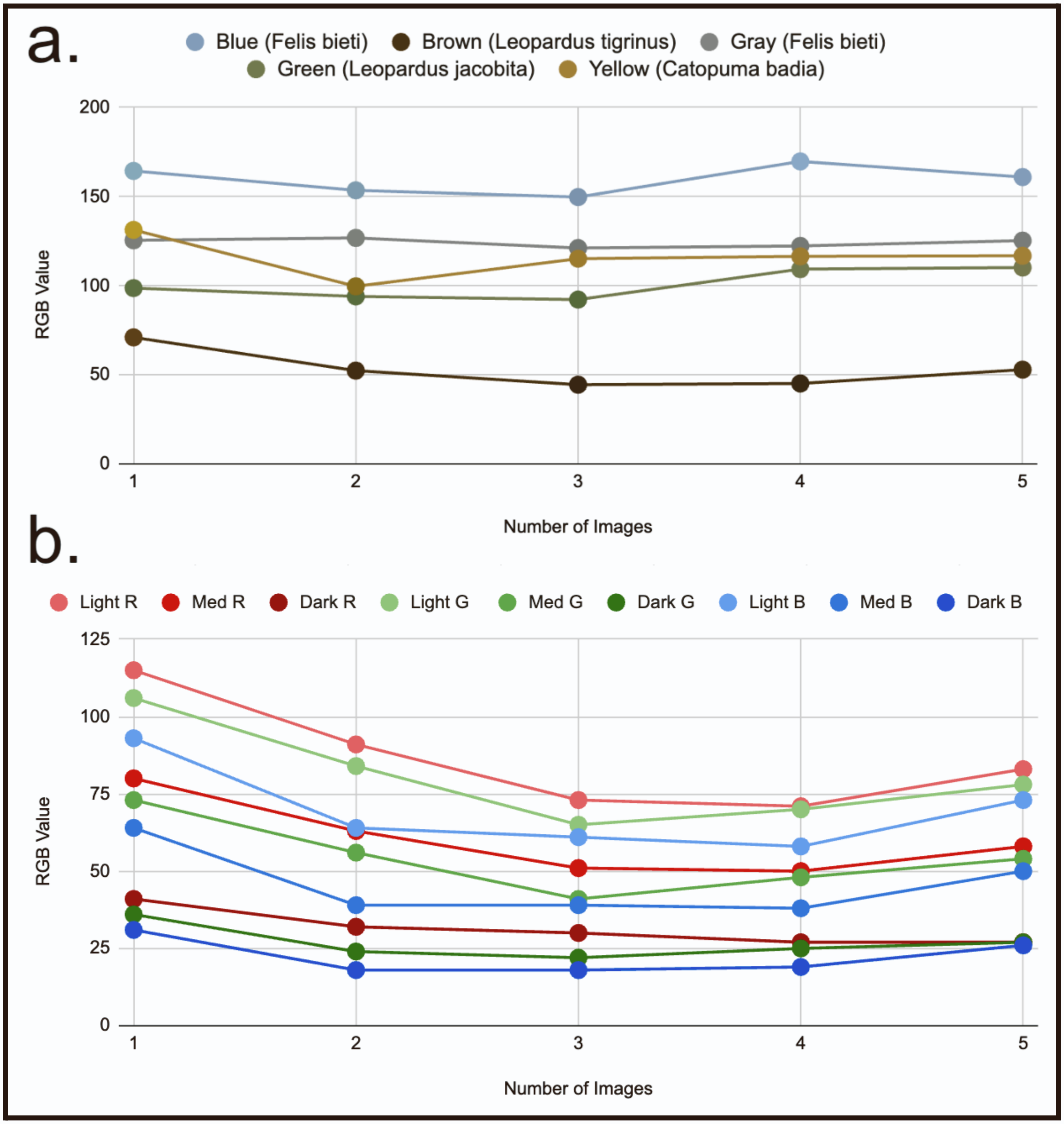

Figure S6: RGB values with increasing numbers of images, separated by species and shade, related to STAR Methods. (a) Average RGB values by the number of images used for representative image sets: all brown-eyed individuals from *Leopardus tigrinus*, all green-eyed individuals from *Leopardus jacobita*, all yellow-eyed individuals from *Catopuma badia*, and all gray- and blue-eyed individuals from *Felis bieti*. RGB values displayed were averages between the R, G, and B values for all shades. Point colors correspond to the average eye color for that number of images. (b) RGB values by the number of images used for the dark, medium, and light shades present in the brown eyes of *Leopardus tigrinus*. The leveling off in both parts of the figure indicates that the sample is sufficient.

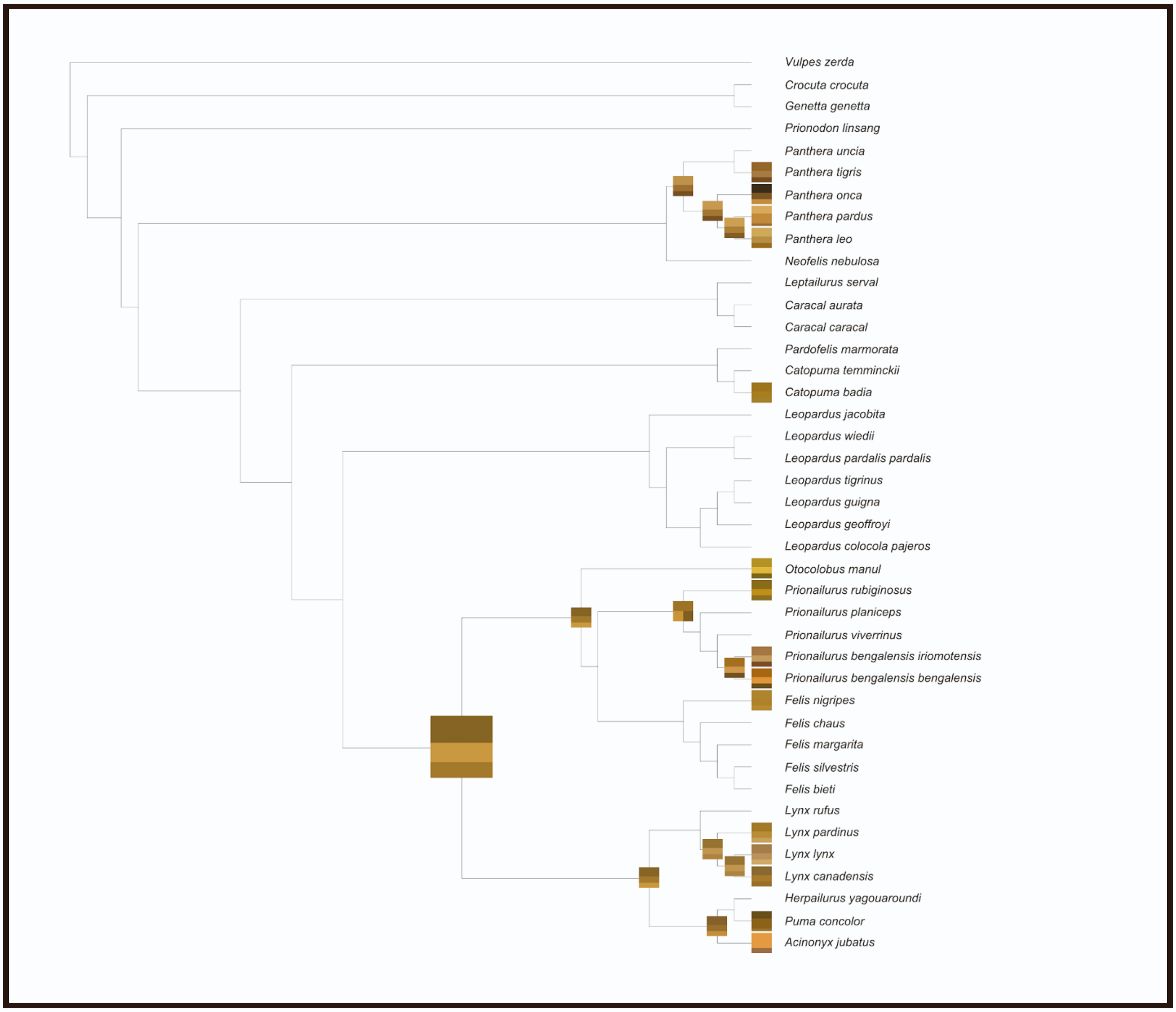

Figure S7: Reconstruction of the ancestral states of the shades of yellow eyes, related to Figure 5. The squares at each node are the quantitative reconstructed shades. The proportion of a square that a shade takes up indicates how common that shade is in the data. Exact branch lengths are not plotted.

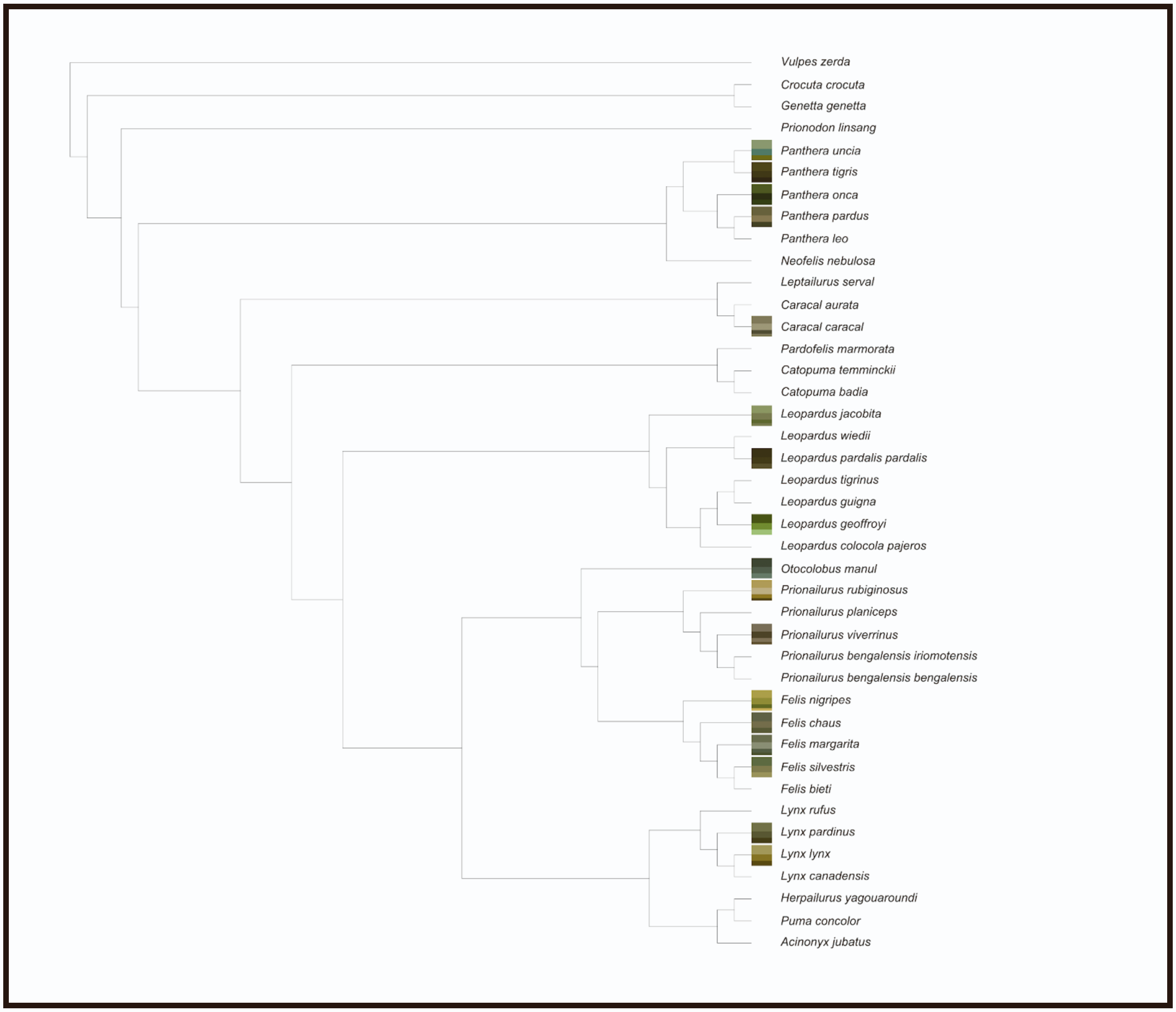

Figure S8: Reconstruction of the ancestral states of the shades of green eyes, related to Figure 5. The squares at each node are the quantitative reconstructed shades. The proportion of a square that a shade takes up indicates how common that shade is in the data. Exact branch lengths are not plotted.

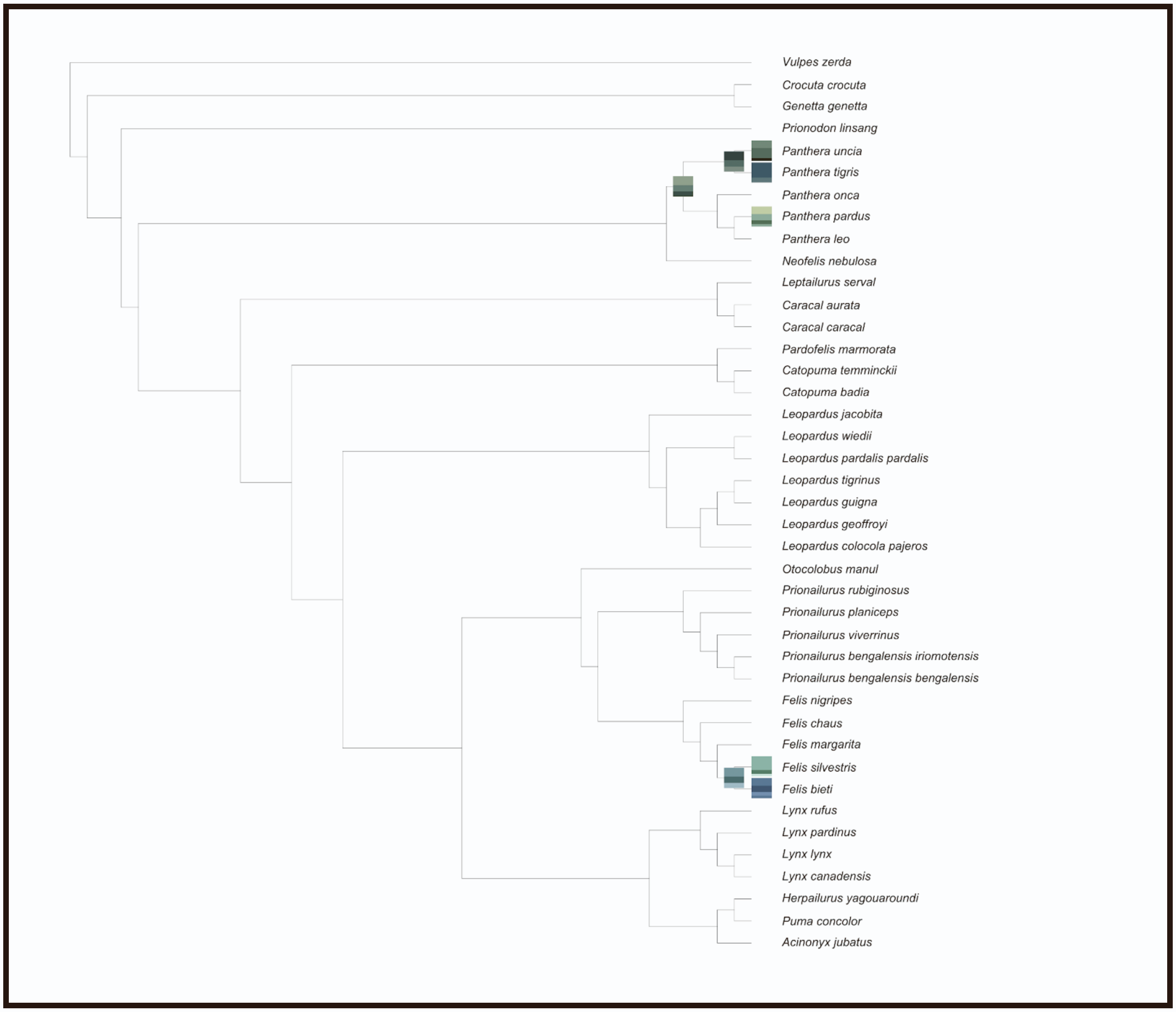

Figure S9: Reconstruction of the ancestral states of the shades of blue eyes, related to Figure 5. The squares at each node are the quantitative reconstructed shades. The proportion of a square that a shade takes up indicates how common that shade is in the data. Exact branch lengths are not plotted.

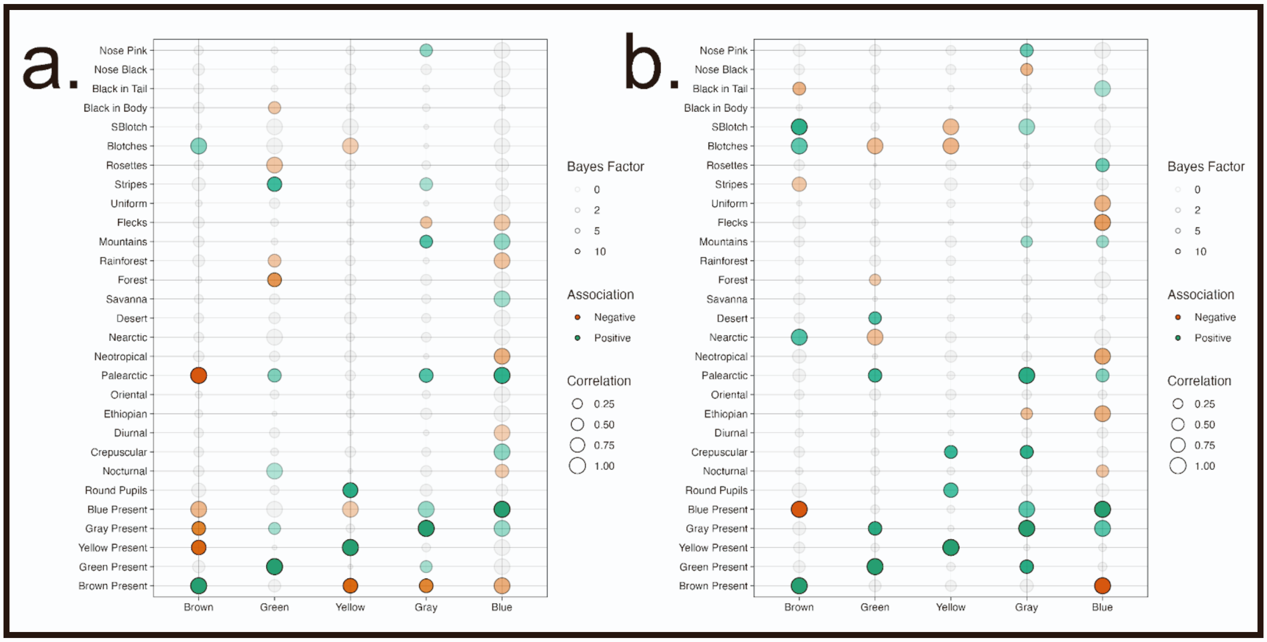

Figure S10: Correlations between the presence of each eye color and various physical, behavioral, and environmental factors for just the most common eye colors (a) and when all of the subspecies were added to the tree (b), related to Figure 6. Larger circles correspond to stronger correlations and more opaque circles correspond to more significant correlations. Green circles have a positive correlation, red circles have a negative correlation, and gray circles do not meet the significance threshold (Bayes factor = 2).

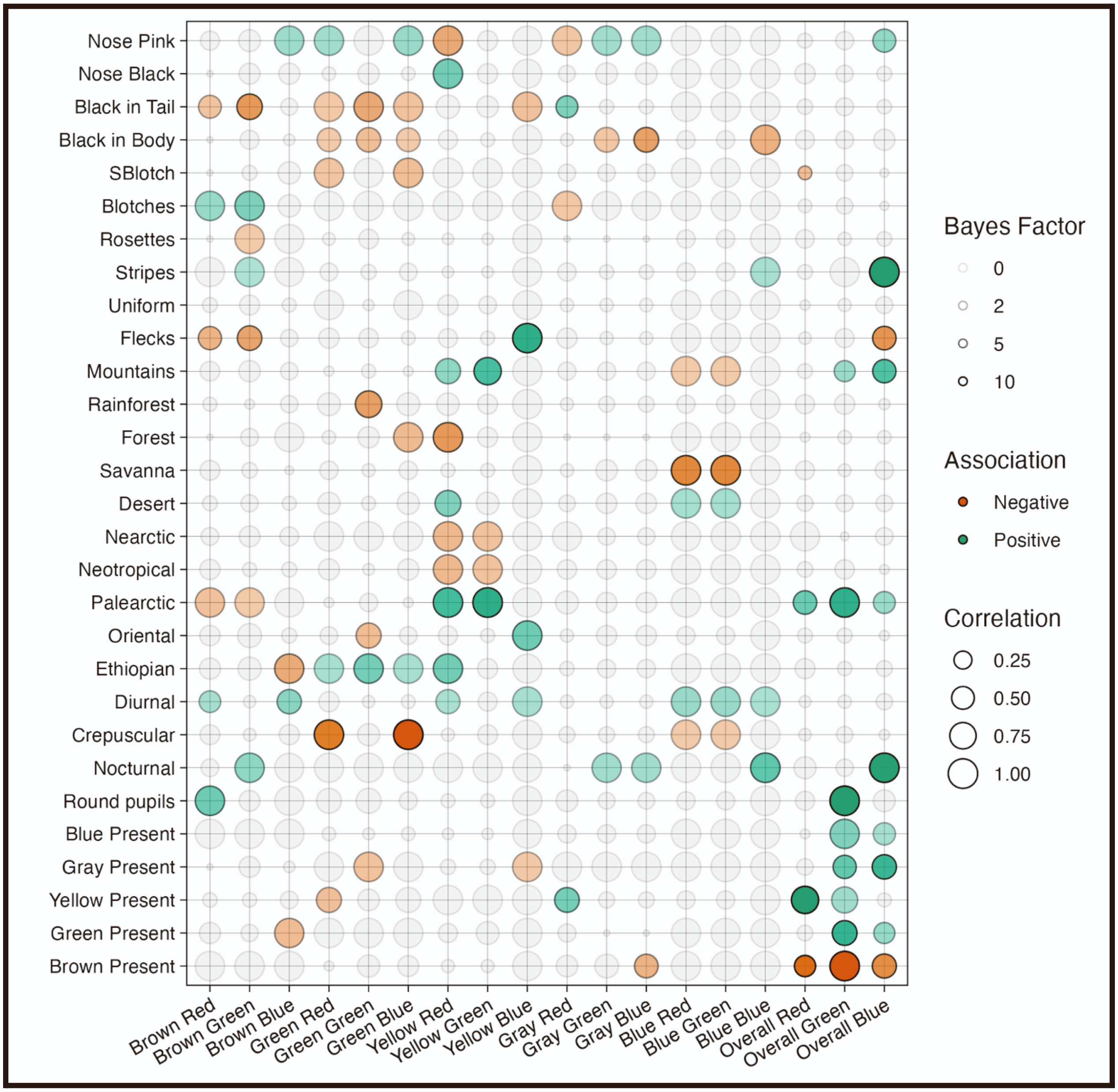

Figure S11: Correlations between the shades of the RGB values of each eye color and various physical, behavioral, and environmental factors, related to Figure 6. Larger circles correspond to stronger correlations and more opaque circles correspond to more significant correlations. Green circles have a positive correlation (i.e. lighter shade), red circles have a negative correlation (i.e. darker shade), and gray circles do not meet the significance threshold (Bayes factor = 2).

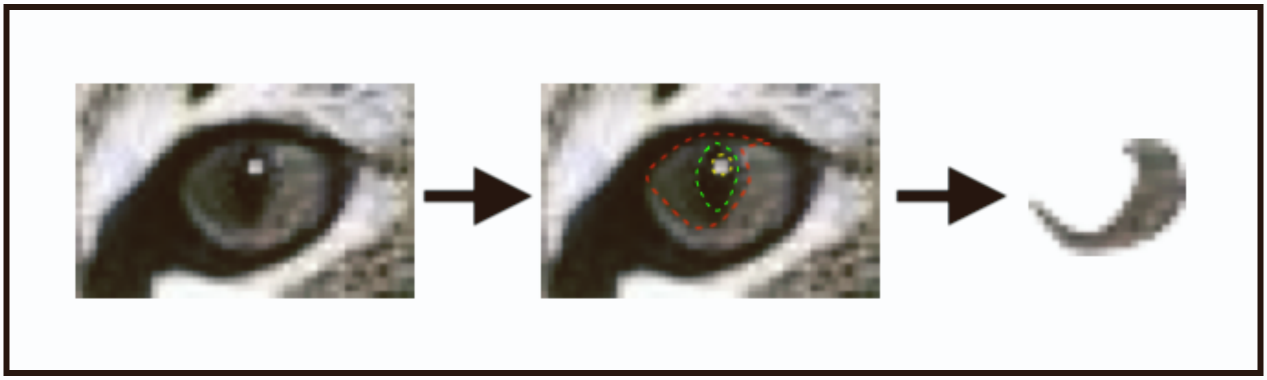

Figure S12: Example of the iris cropping process, related to STAR Methods. On the left is an eye of *Leopardus geoffroyi*, the Geoffroy's cat. In the middle are dotted lines around parts of the image to cut out: red - shadow, green - pupil, yellow - glare. On the right is the resulting iris used for analysis. Credit to dreamstime.com for the original photo.

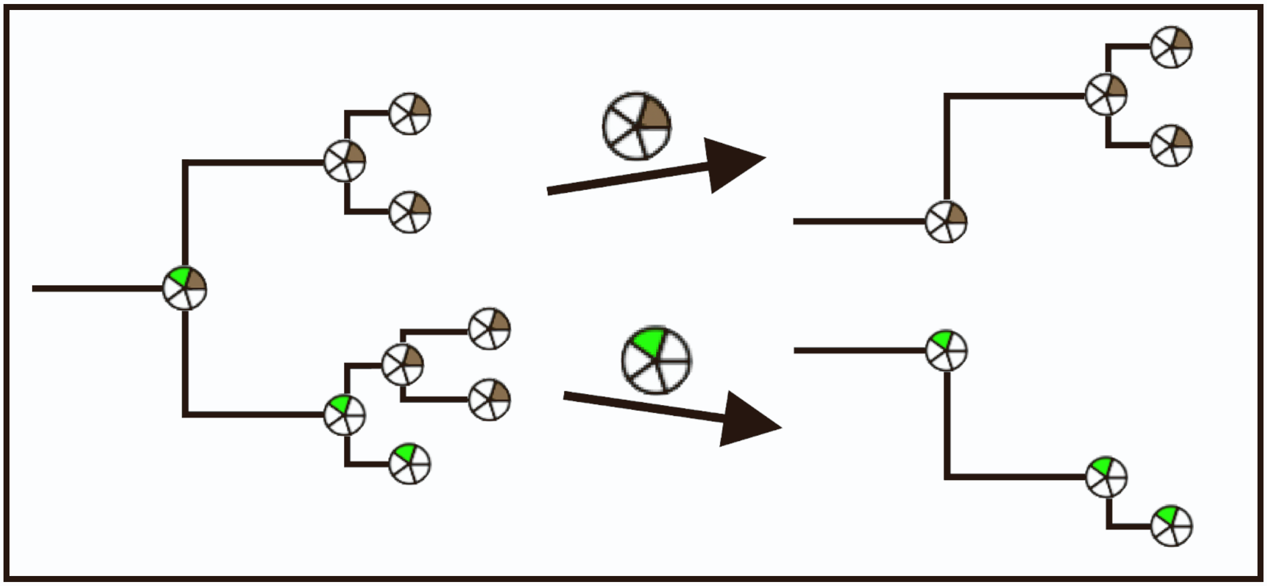

Figure S13: An example phylogenetic tree with illustration of tree separation for shade analysis, related to STAR Methods. The five-wedge pie charts indicate presence (color) or absence (white) of various iris colors. Here, the ancestral node has brown and green eyes. The reconstruction for that node's brown eye shades, shown after the upper arrow, include all the continuous, brown-eyed descendants. The green reconstruction, after the bottom arrow, is done the same way.
